# Spatial analysis of malignant-immune cell interactions in the tumor microenvironment using topological data analysis

**DOI:** 10.1038/s41540-026-00763-7

**Published:** 2026-06-13

**Authors:** Seol Ah Park, Davide Gurnari, Paweł Dłotko, Yongsoo Kim, Jooyoung Hahn

**Affiliations:** 1 Department of Dosimetry and Application of Ionizing Radiation, Faculty of Nuclear Sciences and Physical Engineering, Czech Technical University in Prague, Prague, Czech Republic; 2 Dioscuri Centre in Topological Data Analysis, Institute of Mathematics, Polish Academy of Sciences, Warsaw, Poland; 3 Discouri Center for Topological Data Analysis, Center of Trustworthy AI for Life Sciences - International Research Agendas Programme, University of Warsaw, Warsaw, Poland; 4 Amsterdam UMC, Vrije Universiteit Amsterdam, Department of Pathology, Cancer Center Amsterdam, Amsterdam, The Netherlands; 5 Department of Software Engineering, Faculty of Nuclear Sciences and Physical Engineering, Czech Technical University in Prague, Prague, Czech Republic

**Keywords:** Cancer, Computational biology and bioinformatics, Immunology, Oncology

## Abstract

The spatial interactions between malignant and immune cells in the tumor microenvironment are important for tumor immunobiology and patient outcomes. However, analytical tools that can extract rigorous yet interpretable spatial features and link them to patient outcomes remain limited. We propose a framework integrating TDA with statistical approaches to extract interpretable spatial features characterizing malignant-immune interactions. We introduce Topological Malignant Region (TopMR), which uses topological persistence to automatically define regions of malignant cells, providing an objective reference for spatial analysis even when tumor boundaries are ambiguous. Global-scale infiltration is quantified using signed distances from immune cells to the TopMR boundary and local-scale interactions are captured via malignant cell density around individual immune cells. These global and local features are integrated into a unified signed distance-density ($s{\mathcal{DD}}$) space, enabling comprehensive characterization of spatial patterns. We apply this framework to high-resolution multiplex immunofluorescence images of diffuse large B-cell lymphoma, analyzing both malignant-enriched and tumor border regions. Two-stage hierarchical clustering stratifies patients based on spatial interaction patterns, revealing associations with survival outcomes. This framework provides an end-to-end pipeline from spatial feature extraction to clinical interpretation, suggesting how region-aware spatial analysis can capture biologically meaningful patterns linked to patient survival.

## Introduction

Elucidating the spatial interactions between malignant and immune cells in the tumor microenvironment (TME) is critical for understanding tumor progression and predicting patient outcomes^1,2^. The spatial distribution of these cells reflects complex biological processes such as immune infiltration and evasion, and spatial analysis can help disentangle these processes and link them to clinical prognosis^3,4^. From an analytical perspective, spatial analysis within the TME typically involves quantifying spatial relationships among different cell populations (e.g., tumor and immune cells) and interpreting how these quantified patterns reflect their interactions. Therefore, developing metrics that provide both rigorous quantification and biological interpretability is essential.

Numerous studies have developed methods to extract spatial features from cellular imaging data to correlate these features with patient outcomes. Point-based analyses have quantified tumor-immune organization using cell densities and nearest-neighbor statistics^5^, whereas graph-based models have represented cellular neighborhoods to capture structural patterns and enable patient clustering^6^. Another integrative framework has outlined major categories of spatial analysis, such as localization-, distance-, and graph-based metrics, highlighting their potential clinical relevance^7^. In diffuse large B-cell lymphoma, combining spatial descriptors (e.g., Ripley’s *L*-function) with conventional cell counts and densities has yielded complementary prognostic insights^8^. These approaches predominantly rely on cell-to-cell proximity, capturing local spatial relationships within limited neighborhoods.

Beyond local interactions, some studies have shown that region- and boundary-scale organization, such as tumor-stroma rims or hypoxic cores, can strongly influence immune exclusion, infiltration, and clinical outcomes, sometimes outperforming nearest-neighbor metrics in a context-dependent manner^3,9–11^. Indeed, direct cell-to-cell distances are often biased by local sampling density and structural voids: dense malignant regions yield smaller distances, whereas stromal gaps inflate them. Without accounting for spatial inhomogeneity, such proximity measures may capture sampling artifacts rather than true biological structure^12^. These issues motivate moving beyond local metrics toward mesoscopic, region-aware representations that are both robust and biologically interpretable.

Building on this motivation, recent studies have begun to consider regions of cells to identify spatial interactions between tumor and immune cells. A workflow^13^ characterized spatial distributions of multiple immune markers within pathologist-annotated tissue regions using point pattern statistics and cluster analysis. A statistical framework^14^ extended Ripley’s *K*-function to quantify region-to-cell rather than cell-to-cell interactions by computing distances from tumor-defined regions and applying *K*-function analyses across levelsets. The method effectively showed spatial patterns in histopathological images across patients. Although the tumor region was manually selected by experts, this work showed the potential of region-to-cell approaches for investigating spatial patterns within the TME. Additionally, Topological Data Analysis (TDA)^15–17^ has emerged as a distinct mathematical framework that characterizes spatial organization through topological features rather than conventional statistics. One approach, based on chromatic alpha complexes, quantifies spatial relationships among differently labeled cells by tracking the scale at which shared empty regions appear as virtual cell boundaries expand^18^. Another framework employs Dowker and Witness persistent homology to capture proximity and clustering patterns among synthetic cell populations^19^. Dowker persistence encodes pairwise interactions, whereas Witness persistence treats one population as landmarks to assess the spatial distribution of others. Although not yet applied to biological data, these topology-based approaches may possibly offer a rigorous mathematical framework for quantifying intermixing between cell types.

In this study, we propose a TDA-based integrative framework that combines topological and statistical approaches to extract interpretable spatial interaction features from the TME and relate them to clinical outcomes. Our framework comprises four components, each designed to address a key limitation of traditional spatial analyses: (1) we define a *Topological Malignant Region* (TopMR) to reconstruct the global organization of malignant cells, providing a topologically defined tumor regions even when explicit margins are absent or irregular; (2) we extract global and local interaction features—global features quantify immune infiltration through signed distances from the TopMR and local features capture malignant cell density around individual immune cells, reflecting micro-scale heterogeneity; (3) we employ *Ball Mapper*^20^ to summarize the resulting high-dimensional interaction space, enabling visualization and micro-clusters of spatial patterns across all datasets; and (4) we link these summarized interaction patterns to patient survival using a two-stage hierarchical clustering approach that accounts for varying numbers of data samples per patient and automates parameter selection. Together, these components combine topological abstraction, quantitative analysis, and biological interpretability to offer a coherent approach for characterizing tumor-immune interaction with potential clinical relevance. Finally, we apply our framework to point cloud data derived from high-resolution multiplex immunofluorescence (mIF) images of diffuse large B-cell lymphoma (DLBCL), annotated as either malignant cell-enriched or tumor border regions exhibiting distinct spatial organizations. TopMR robustly captures diverse global morphologies of malignant cell organization across both image types. By integrating global and local interaction features, our framework captures distinct spatial interaction patterns that are indicative of differences in patient survival outcomes.

## Results

Building on the framework outlined in the Introduction, the following five subsections develop and present each of its components in turn. The framework operates on point clouds extracted from multiplex immunofluorescence (mIF) images of primary central nervous system diffuse large B-cell lymphoma (DLBCL); the data acquisition, segmentation, pathologist annotation, and phenotype aggregation rationale are described in the Methods section. Here, we begin by formalizing the input as the mathematical objects used throughout the methodological development that follows. Let us denote malignant cell’s (PAX5^+^) and the *i*^th^ aggregated immune cell’s locations as point clouds ${\mathcal{R}}$ and ${{\mathcal{Q}}}^{i}$ in a mIF image (Ω), respectively:

1 $${\mathcal{R}}=\{{\bf{r}}\in \Omega \subset {{\mathbb{R}}}^{2}:{\bf{r}}\,{\rm{is\; a\; location\; of\; malignant\; cell}}\},$$

2 $$\begin{array}{l}{{\mathcal{Q}}}^{i}=\left\{{{\bf{q}}}^{i}\in \Omega \subset {{\mathbb{R}}}^{2}:{{\bf{q}}}^{i}\,{\rm{is}}\,{\rm{a}}\,{\rm{location}}\,{\rm{of}}\,{\rm{immune}}\,{\rm{cells}}\,{\rm{of}}\right.\\ \left.{\rm{the}}\,{i}^{{\rm{th}}}\,{\rm{aggregated}}\,{\rm{phenotype}}\right\}.\end{array}$$

where

3 $$i\in {\mathcal{I}}=\{{{\rm{CD3}}}^{+}{{\rm{CD8}}}^{-},{{\rm{CD3}}}^{+}{{\rm{CD8}}}^{+},{{\rm{CD163}}}^{+}\}.$$

Throughout the following subsections, ${\mathcal{R}}$ serves as the reference population for region reconstruction, while ${{\mathcal{Q}}}^{i}$ provides the immune cells whose spatial relationship to that region we quantify.

### TopMR captures global malignant architecture and yields interpretable signed distances

**Fig. 1:**
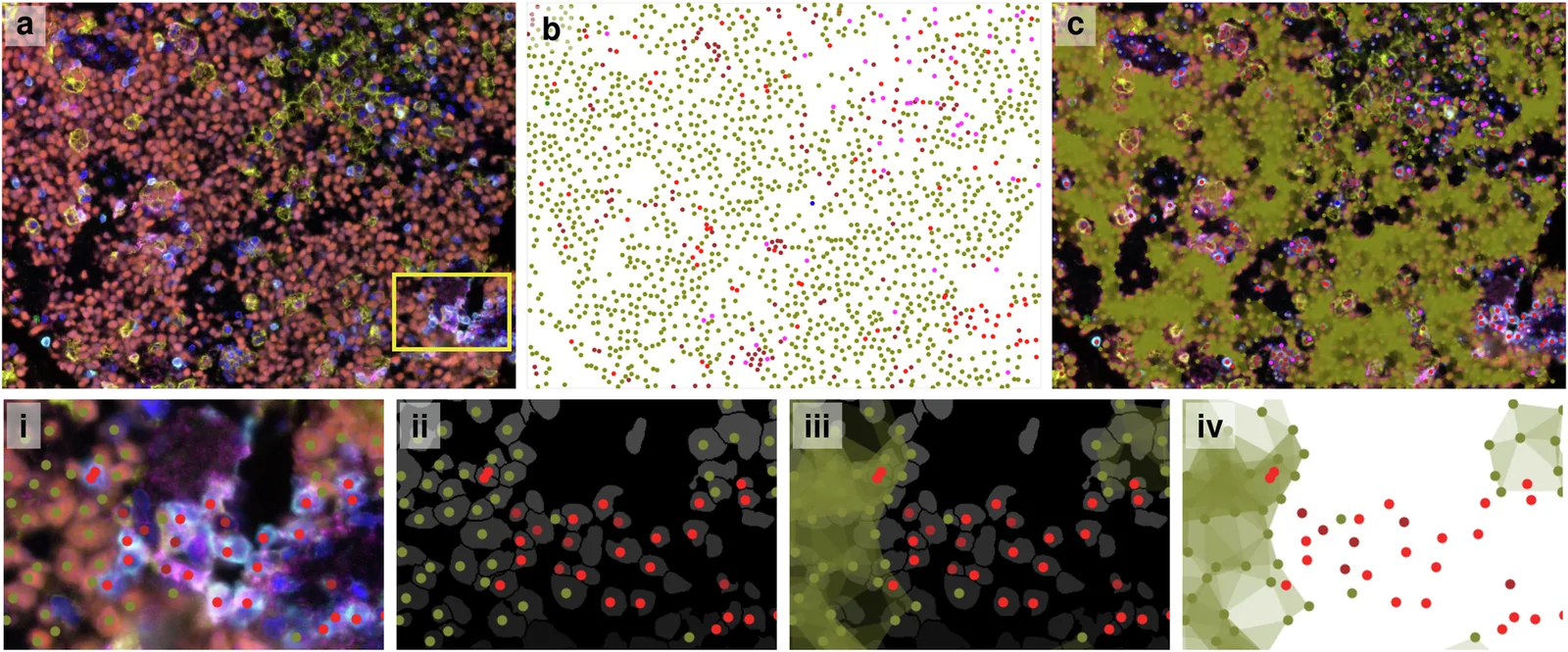
Topological malignant region (TopMR): global spatial shapes of malignant cell distributions. **a** A raw multiplex immunofluorescence (source:^8^). The highlighted area by the yellow rectangle is presented with more details in the second row. **b** Point clouds of cell centers colored by cell type: olive (malignant), red (CD3^+^CD8^−^PD-1^−^), blue (CD3^+^CD8^−^PD-1^+^), magenta (CD163^+^PD-L1^−^), and brown (CD163^+^PD-L1^+^) (see Table 1). **c** TopMR (olive region) overlaid on (**a**) demonstrates global spatial shapes of malignant cell distributions in (**a**) and (b). (i) illustrates different phenotypes on yellow box in (**a**). (ii) shows the segmented cells. (iii) presents the TopMR on (ii). (iv) illustrates the spatial context between immune cells and TopMR. Image segmentation and phenotyping were performed using InForm software (Akoya Biosciences).

We first define a topology-based representation of the global organization of ${\mathcal{R}}$, which we call the Topological Malignant Region (TopMR) (e.g. Fig. 1), and we then show that the construction adapts robustly across diverse spatial configurations of malignant cells.

Malignant and immune cells in tissue are spatially non-random due to underlying biological architecture. Structural components such as necrotic zones, stromal regions, vascular lumens, and non-malignant compartments often create spatial voids or clustered distributions (Figs. 3 and 5). We therefore propose a method to capture underlying spatial organization from the given point cloud, providing robustness against heterogeneous or irregular cell distributions. It enables the analysis to focus on biologically meaningful malignant-immune interactions rather than structural biases.

To extract the spatial organization, we employ tools from TDA. In a Vietoris-Rips filtration, a distance threshold is gradually increased, and all pairs of points closer than this threshold are connected by an edge. Whenever every pair within a group of points lies within the threshold, the group forms a simplex: an edge for two points, a triangle for three, a tetrahedron for four, and so on. The collection of all such simplices is a simplicial complex, which generalizes a network by recording not only pairwise connections (edges) but also higher-order relationships among points that are all pairwise within the threshold. Because the construction depends only on pairwise distances, it promotes connectivity and provides robust global shape extraction. As the threshold grows, topological structures such as connected components (*H*_0_) and loops (*H*_1_) emerge and subsequently disappear (e.g., components merge and loops are filled in by higher-dimensional simplices). This entire process is summarized in a persistence diagram (PD), which records the birth and death of each such structure as a function of the distance threshold^15,16^.

Small perturbations in point positions produce only small changes in pairwise distances. Therefore, birth and death values shift only slightly, making PDs stable under positional noise such as cell-center detection, segmentation errors, and a small fraction of phenotype misclassifications. Structures with long lifetimes (large gap between birth and death) can represent robust spatial patterns of the point cloud, whereas short-lived structures near the diagonal of the PD typically reflect noise or local irregularities. Ideally, the PD should cover all scales, but this becomes computationally challenging for images with a large number of points^21^. Hence, we set a practical limit on the maximum length scale:

4 $${\ell }_{\max }=C{\left(\frac{| \Omega | }{| {\mathcal{R}}| }\right)}^{\frac{1}{2}},$$

where $| {\mathcal{R}}|$ is the number of points in ${\mathcal{R}}$ (Eq. (1)), ∣Ω∣ is the area of the mIF image domain Ω, and *C* = 3. This scale, derived from the square root of the inverse point density, adapts to each image to account for variation in malignant cell counts. Such an adaptive approach ensures computational efficiency across images with differing cell counts. The constant *C* is chosen to balance computational efficiency with the ability to capture meaningful topological features. If *C* is too small, the number of generated simplices is insufficient to reflect relevant structures. On the other hand, if *C* is too large, it connects nearly all points, resulting in high computational cost and reduced biological interpretability. In this study, the value of *C* was determined empirically based on practical performance considerations and observed performance.

Now, we obtain the PD using ${\ell }_{\max }$ (Eq. (4)). Each point (*b*, *d*) in the PD marks the birth and death of a topological feature. We select the most persistent feature (the dominant topological structure in ${\mathcal{R}}$), that is, the point in *H*_1_ with the longest finite lifetime, given by

5 $$({b}_{m},{d}_{m})=\mathop{\arg \,\max }\limits_{(b,d)\in {\mathcal{P}}}| d-b| .$$

**Fig. 2:**
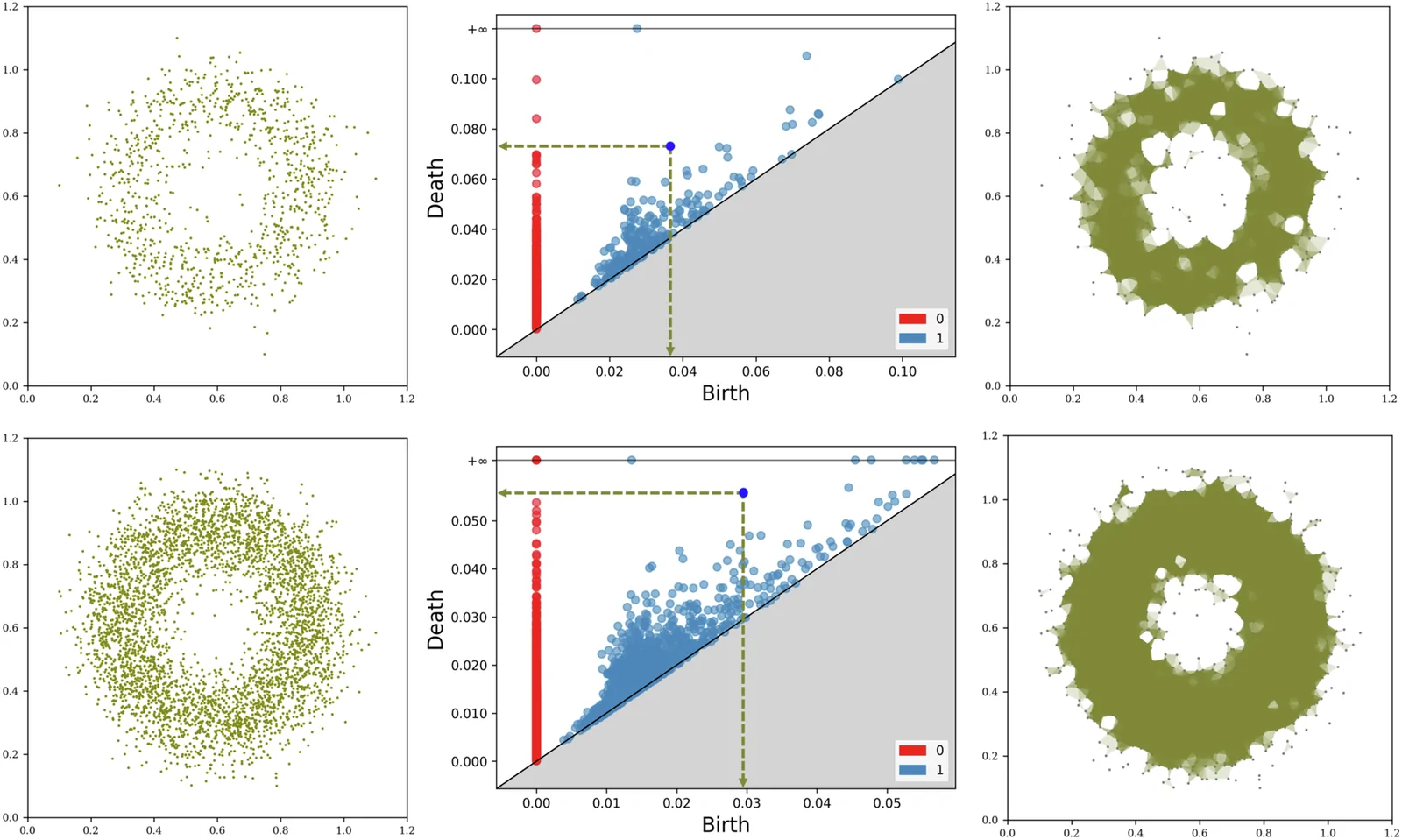
Persistence diagrams and TopMR for point clouds with density difference. Point clouds (left column) with 1 ⋅ 10^3^ (top) and 4 ⋅ 10^3^ (bottom) points are in the same domain. The corresponding persistence diagrams (middle colume) are presented by the maximum length scale in Eq. (4), ${\ell }_{\max }=1.14\cdot 1{0}^{-1}$ (top) and 5.69 ⋅ 10^−2^ (bottom). From the most persistent feature (Eq. (5)), (3.66 ⋅ 10^−2^, 7.30 ⋅ 10^−2^) (top) and (2.95 ⋅ 10^−2^, 5.58 ⋅ 10^−2^) (bottom), the characteristic length *ℓ*_chr_ (Eq. (6)) is calculated with *ℓ*_cll_ = *∞* in these examples. The corresponding TopMR (right column) is visualized, which robustly shows an annulus shape.

Motivated by the idea that the shape corresponding to the longest finite lifetime in the PD reflects the most persistent topological structure of the distribution, we take the midpoint between *b*_*m*_ and *d*_*m*_ (Fig. 2). This value is constrained by a biologically relevant upper bound, ${\ell }_{{\rm{cll}}}=\sqrt{2}$ Ø, (Eq. (6)), because long-range connections between malignant cells lack biological plausibility. Given the average malignant cell diameter of ~25 μm in DLBCL^22^, we set Ø ≈ 50 μm to account for potential segmentation errors in cell center detection. Also, Ø ≈ 50 μm was found as the most informative short-range interaction length in the previous study^8^.

Finally, using the characteristic length,

6 $${\ell }_{{\rm{chr}}}=\min \left(\frac{{b}_{m}+{d}_{m}}{2},{\ell }_{{\rm{cll}}}\right).$$

we reconstruct the global spatial shape of malignant cells by the union of 2D simplices (triangles), which we refer to as TopMR. Figure 1 demonstrates that TopMR captures the global spatial shape of malignant cells. The second row (yellow rectangle) shows that while most immune cells (CD3^+^CD8^−^PD-1^−^ and CD163^+^PD-L1^+^) lie outside TopMR, some immune cells infiltrate into TopMR (panel (iv) in Fig. 1), which will be quantified in terms of malignant-immune interactions in the subsections that follow.

**Fig. 3:**
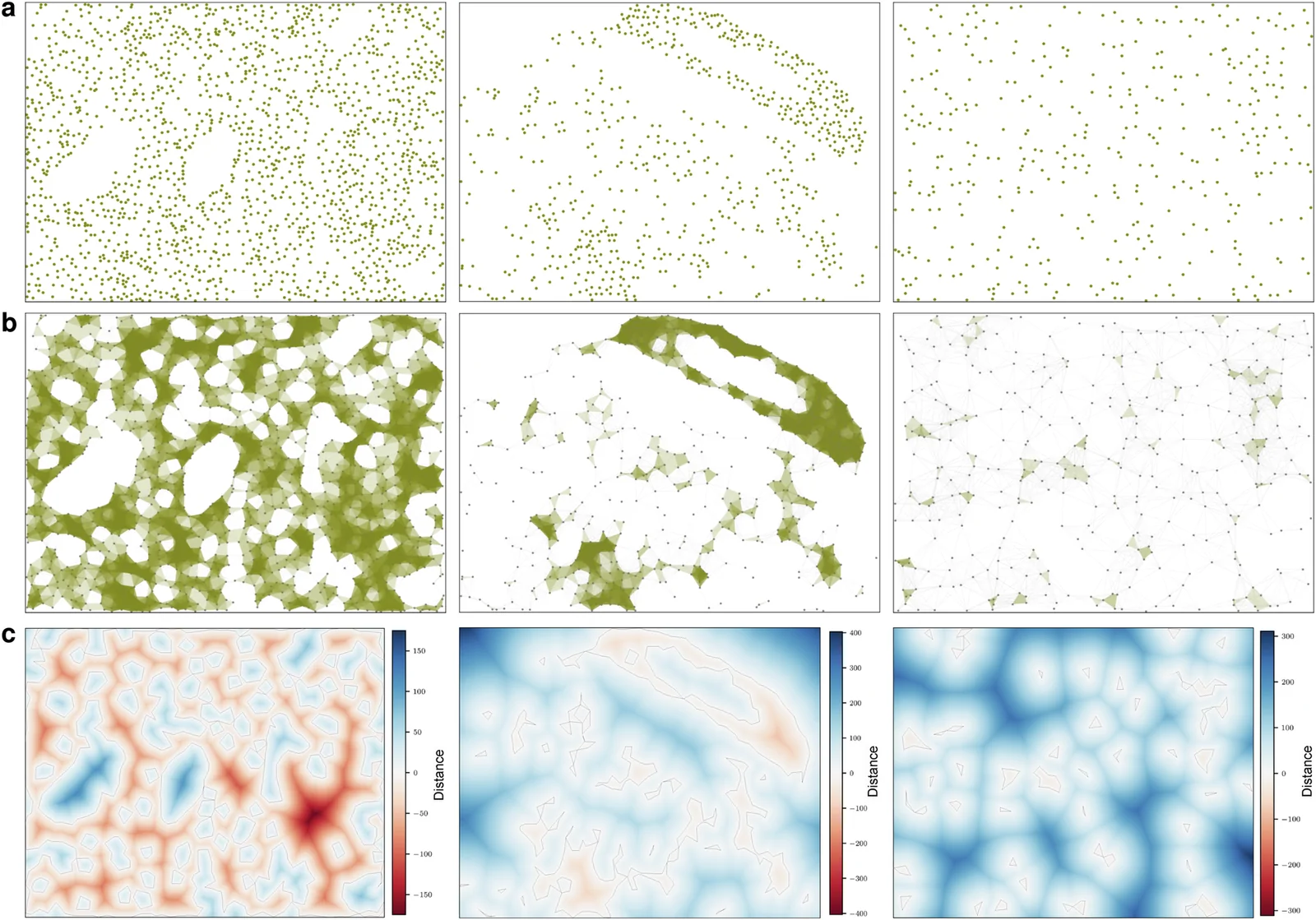
TopMR and signed distance functions across diverse malignant cell distributions. Each column shows a distinct spatial configuration of malignant cells (**a**), the corresponding TopMR (**b**), and the resulting signed distance function (**c**). For better visualization of the TopMR boundary (where the signed distance is zero), the color scale is set symmetrically for positive and negative values. Red and blue indicate negative and positive distances, respectively, highlighting regions inside (infiltrated) and outside (not infiltrated) the TopMR; its boundary is shown in gray.

To examine how TopMR responds to qualitatively distinct malignant cell distributions, we apply the construction above to representative DLBCL examples. We present how the proposed TopMR effectively captures the global spatial structure of malignant cell distributions and provides meaningful signed distance functions that reflect spatial interaction patterns. In the first column of Fig. 3, malignant cells appear generally dense but contain several rounded voids, regions where no malignant cells are present. The corresponding TopMR successfully captures the overall spatial shape, considering these voids. The signed distance function shows high positive values inside these voids, indicating that immune cells located there are far from malignant region, interpretable as regions of weak infiltration. The second column shows a more complex case, where malignant cells form a ring-like structure with a non-uniform spatial distribution. Some areas are densely populated while others are sparse. The resulting TopMR identifies the global ring pattern and extract only the most concentrated regions into large area of TopMR. The signed distance function from this TopMR boundary reflects these variations, indicating that distance computations remain robust even under highly non-uniform distributions. In the third column, malignant cells are sparsely scattered with no prominent structure. In this case, the TopMR captures only small and isolated regions. The corresponding signed distance function shows small negative values, suggesting weak spatial attraction to the malignant regions. Overall, these results illustrate that TopMR effectively captures diverse spatial patterns such as dense with voids, non-uniform density, or sparse. Also it provides interpretable signed distance function that reflect immune cell infiltration relative to the malignant region defined by their configuration.

### Signed distance-density space quantifies malignant-immune interaction patterns

With TopMR providing a region-level reference for ${\mathcal{R}}$, we now construct the spatial features that quantify malignant-immune interactions. We define complementary global and local features, the signed distance from the TopMR boundary and the local malignant cell density around each immune cell, and combine them into a unified feature space and an image-level descriptor that will serve as input to the topological summarization presented later.

#### Global feature: computing signed distance fields from TopMR boundaries

In the TME, immune cell positioning can be influenced by signaling gradients that emerge from the collective spatial arrangement of malignant cells can strongly influence immune cell positioning^23,24^. This motivates a region-to-cell (malignant region) perspective, rather than a purely cell-to-cell (e.g., nearest-neighbor) view, for capturing these relationships more robustly. Accordingly, we quantify malignant-immune spatial interactions using *signed distances* from the TopMR boundary to each immune cell (TopMR-to-cell), since TopMR captures regions of malignant cells.

Denoting TopMR as the region ${\mathcal{T}}$, we compute the distance function *d*(**x**) from TopMR boundary ($\partial {\mathcal{T}}$) using the eikonal equation ^25^. In order to quantify the infiltration of immune cells ${{\mathcal{Q}}}^{i}$, we measure a signed distance:

7 $$\varphi ({\bf{x}})=\left\{\begin{array}{ll}-d({\bf{x}}),\, & \,{\rm{if}}\,\,\,\,\,{\bf{x}}\in {\mathcal{T}},\\ d({\bf{x}}),\, & \,{\rm{if}}\,\,\,\,\,{\bf{x}}\notin {\mathcal{T}},\\ 0,\, & \,{\rm{if}}\,\,\,\,\,{\bf{x}}\in \partial {\mathcal{T}}.\end{array}\right.$$

Note that *φ*(**x**) is negative if the cell is inside TopMR (infiltration or potential attraction) and positive if outside (no infiltration).

#### Local feature: estimating local malignant cell density around immune cells

We also compute local malignant cell density to distinguish cases where immune cells have similar infiltration levels into the TopMR but different surrounding malignant cell counts. A Gaussian function is used on the square *W*_*ω*_(**r**) (side: *ω* = 4 Ø, center: ${\bf{r}}\in {\mathcal{R}}$):

8 $${G}_{{\bf{r}}}({\bf{x}})=\left\{\begin{array}{ll}c{g}_{{\bf{r}}}({\bf{x}})=c\exp \left(-\frac{| {\bf{x}}-{\bf{r}}{| }^{2}}{2{\sigma }^{2}}\right),\, & {\bf{x}}\in {W}_{\omega }({\bf{r}}),\\ 0,\, & {\bf{x}}\notin {W}_{\omega }({\bf{r}}),\end{array}\right.$$

where *σ* = 0.2*ω* and *c*^−1^ is a constant ${\int }_{{W}_{\omega }({\bf{r}})}{g}_{{\bf{r}}}({\bf{x}})dA$, normalizing over the square. It ensures that the total weight of the Gaussian function over the square equals 1, making *G*_**r**_(**x**) a probability density function. Now, we compute $\bar{\psi }({\bf{x}})=\left({K}_{\sigma }* {\sum }_{{\bf{r}}\in {\mathcal{R}}}{G}_{{\bf{r}}}\right)({\bf{x}})$, where *K*_*σ*_ is the Gaussian kernel with the standard deviation *σ* and the convolution operation (*). We then rescale $\bar{\psi }({\bf{x}})$ to match the range of the signed distance *φ*(**x**), ensuring that both features contribute equally. From the global maximum (*φ*^M^) and minimum (*φ*^m^) of *φ*(**q**^*i*^) and the global maximum (${\bar{\psi }}^{{\rm{M}}}$) of $\bar{\psi }({{\bf{q}}}^{i})$ over all ${{\bf{q}}}^{i}\in {{\mathcal{Q}}}^{i}$ and $i\in {\mathcal{I}}$ across all mIF images, the density function is defined by

9 $$\psi ({\bf{x}})=\left(\frac{{\varphi }^{{\rm{M}}}-{\varphi }^{{\rm{m}}}}{2{\bar{\psi }}^{{\rm{M}}}}\right)\bar{\psi }({\bf{x}}).$$

#### Constructing the signed distance-density ($s{\mathcal{DD}}$) space and image-level features

To quantify malignant-immune cell interactions using both global and local features, we define the signed distance-density $s{\mathcal{DD}}$ space, for $i\in {\mathcal{I}}$ in Eq. (3):

10 $$s{{\mathcal{DD}}}^{i}=\{(\varphi ({{\bf{q}}}^{i}),\psi ({{\bf{q}}}^{i}))\in {{\mathbb{R}}}^{2}:{{\bf{q}}}^{i}\in {{\mathcal{Q}}}^{i}\},$$

where *φ*(**q**^*i*^) and *ψ*(**q**^*i*^) denote the signed distance and density functions, respectively, for each immune cell of the aggregated phenotype **q**^*i*^. These measures are complementary yet independent, capturing distinct aspects of malignant-immune interactions in the subsection $s{\mathcal{DD}}$ features distinguish intermixed, segregated and partially mixed spatial patterns. While signed distance indicates an immune cell’s position relative to the TopMR boundary, the density reflects the local concentration of malignant cells around the immune cell.

To summarize patterns in $s{{\mathcal{DD}}}^{i}$, we apply principal component analysis (PCA), which is parameter-free and yields a fixed set of features from the first two principal components. This also brings interpretable spatial features with stability: small changes in $s{{\mathcal{DD}}}^{i}$ produce only small changes in the PCA features, providing an ideal input for subsequent analysis of summarizing interaction patterns with with Ball Mapper graphs.

From the PCA output, we define the following row vector:

11 $${{\bf{F}}}^{i}=[{{\bf{m}}}^{i},{{\bf{e}}}_{1}^{i},{{\bf{e}}}_{2}^{i}]\in {{\mathbb{R}}}^{6},\,i\in {\mathcal{I}},$$

where ${{\bf{m}}}^{i}=({m}_{h}^{i},{m}_{v}^{i})$, ${{\bf{e}}}_{1}^{i}=({e}_{1h}^{i},{e}_{1v}^{i})$, and ${{\bf{e}}}_{2}^{i}=({e}_{2h}^{i},{e}_{2v}^{i})$ are 2D vectors; averages of signed distance and density values in $s{{\mathcal{DD}}}^{i}$ space, the first and the second principle components of PCA, respectively. For the *n*th image, we construct a feature vector by concatenating the three row vectors:

12 $${{\bf{F}}}_{n}=\left[{{\bf{F}}}^{{{\rm{CD3}}}^{+}{{\rm{CD8}}}^{-}},{{\bf{F}}}^{{{\rm{CD3}}}^{+}{{\rm{CD8}}}^{+}},{{\bf{F}}}^{{{\rm{CD163}}}^{+}}\right]\in {{\mathbb{R}}}^{18}.$$

The resulting vectors summarize the spatial characteristics of malignant-immune interactions and are used for grouping and comparison across images. For clinical interpretation, we later return to the full set of immune phenotypes in the subsection Spatial interaction patterns in $s{\mathcal{DD}}$ space associate with patient survival. We include an image only if ∣*Q*^*i*^∣≥2 for all $i\in {\mathcal{I}}$. Based on this, 972 out of 1337 images are analyzed (687 TR and 285 BR images). Stacking the image-level row vectors vertically yields the feature matrix:

13 $${\bf{F}}=\left[\begin{array}{c}{{\bf{F}}}_{1}\\ {{\bf{F}}}_{2}\\ \vdots \\ {{\bf{F}}}_{972}\end{array}\right]\in {{\mathbb{R}}}^{972\times 18}.$$

**Fig. 4:**
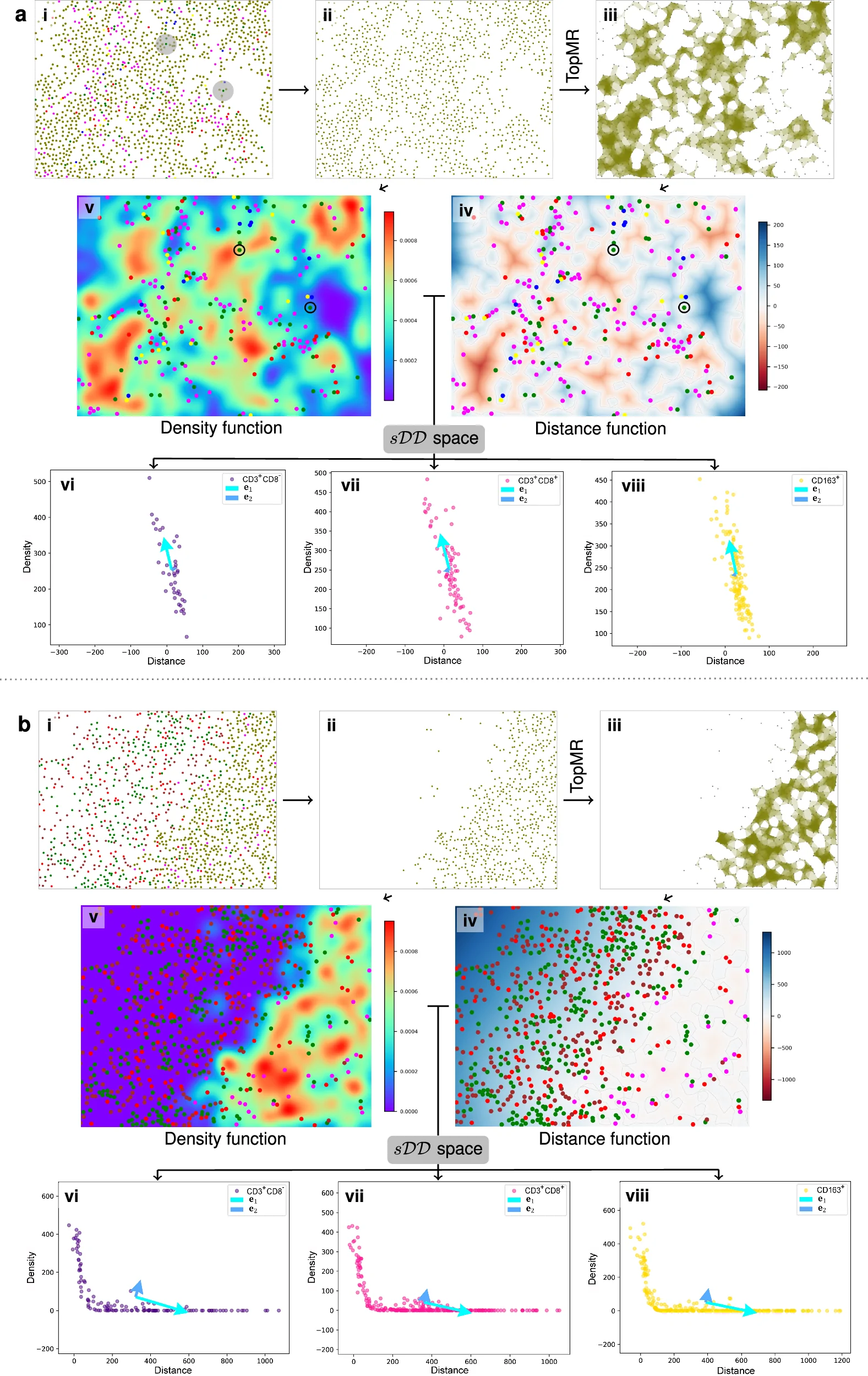
Distance and density functions of point clouds from images. **a** and BR image (**b**): Panels (i)–(v) show analysis using all cell phenotypes, while (vi)–(viii) use aggregated immune phenotypes (see Table 1 for cell type colors). (i) The original point cloud in TR image. (ii) Malignant cell distributions. (iii) TopMR from (ii). (iv) Signed distance function (immune cells overlaid), illustrating immune infiltration (red = inside TopMR, blue = outside). (v) Malignant cell density function around immune cells (purple-to-scarlet colormap for low to high density). (Two example immune cells are highlighted in gray in (i) and circled in (iv)–(v); see main text for details.) (vi)–(viii) $s{{\mathcal{DD}}}^{i}$ spaces (Eq. (10)) for each aggregated immune phenotype $i\in {\mathcal{I}}$ (Eq. (3)). Vectors ${{\bf{e}}}_{1}^{i}$ and ${{\bf{e}}}_{2}^{i}$ are drawn at **m**^*i*^ (Eq. (11)) in each $s{{\mathcal{DD}}}^{i}$ space, visualizing the corresponding 18*D* feature from Eq. (12).

We now show the resulting $s{\mathcal{DD}}$ construction on two representative DLBCL images, contrasting a malignant-enriched (TR) and a tumor-border (BR) configuration. We describe the steps of constructing the $s{\mathcal{DD}}$ space using DLBCL data. Starting from point clouds, we generate the TopMR, compute the signed distance and local density functions, and build the $s{\mathcal{DD}}$ space. We then present this process using two representative examples: TR image (Fig. 4a) and BR image (Fig. 4b). These examples are selected to illustrate how $s{\mathcal{DD}}$ captures spatial malignant-immune interactions for contrasting spatial contexts. Figure 4a shows densely packed malignant cells with immune cells located near the TopMR. Figure 4b shows a sparser, more inhomogeneous malignant distribution, with immune cells positioned farther from the TopMR.

The signed distance functions in Fig. 4a(iv), b(iv) illustrate the spatial positioning of immune cells relative to the TopMR boundary. Red indicates negative values (deeper infiltration), blue indicates positive values (large distance), and white denotes proximity to the boundary. This spatial information is complemented by the local density of malignant cells around immune cells (purple-to-scarlet color bar) where scarlet signifies high malignant cell density. Although they are often correlated, discrepancies can arise; immune cells at similar distances from the TopMR boundary may have different local malignant cell densities. For example, in Fig. 4a(i), two CD8^+^ T cells (PD-1^−^) (small gray disks), lie near the TopMR boundary as shown in the panel (a)-(iv) (black circles). However, they exhibit distinct density values as shown in the panel (a)-(v). This highlights how these measures provide a complementary view of malignant-immune cell interactions.

We aggregate immune cell phenotypes when constructing the $s{\mathcal{DD}}$ space. The resulting $s{\mathcal{DD}}$ patterns for two distinct malignant cell configurations are presented on the panels (vi), (vii), and (viii) in Fig. 4a, b. TR image shows a strong interaction between immune and malignant cells, reflected as a nearly linear pattern. Conversely, BR image shows that immune cells appear enriched at larger distances from the TopMR with sparse malignant cells nearby, corresponding to an exponential-decay pattern. Both patterns can be effectively described by ${{\bf{e}}}_{1}^{i}$ obtained from PCA in Eq. (11): vectors pointing toward the top-left indicate strong interactions (typical of TR images), while vectors pointing toward the bottom-right imply weak interactions (typical of BR images). However, this interpretation does not always hold for all TR and BR images because cell distribution can change continuously to diverse and atypical configurations.

### $s{\mathcal{DD}}$ features distinguish intermixed, segregated and partially mixed spatial patterns

**Fig. 5:**
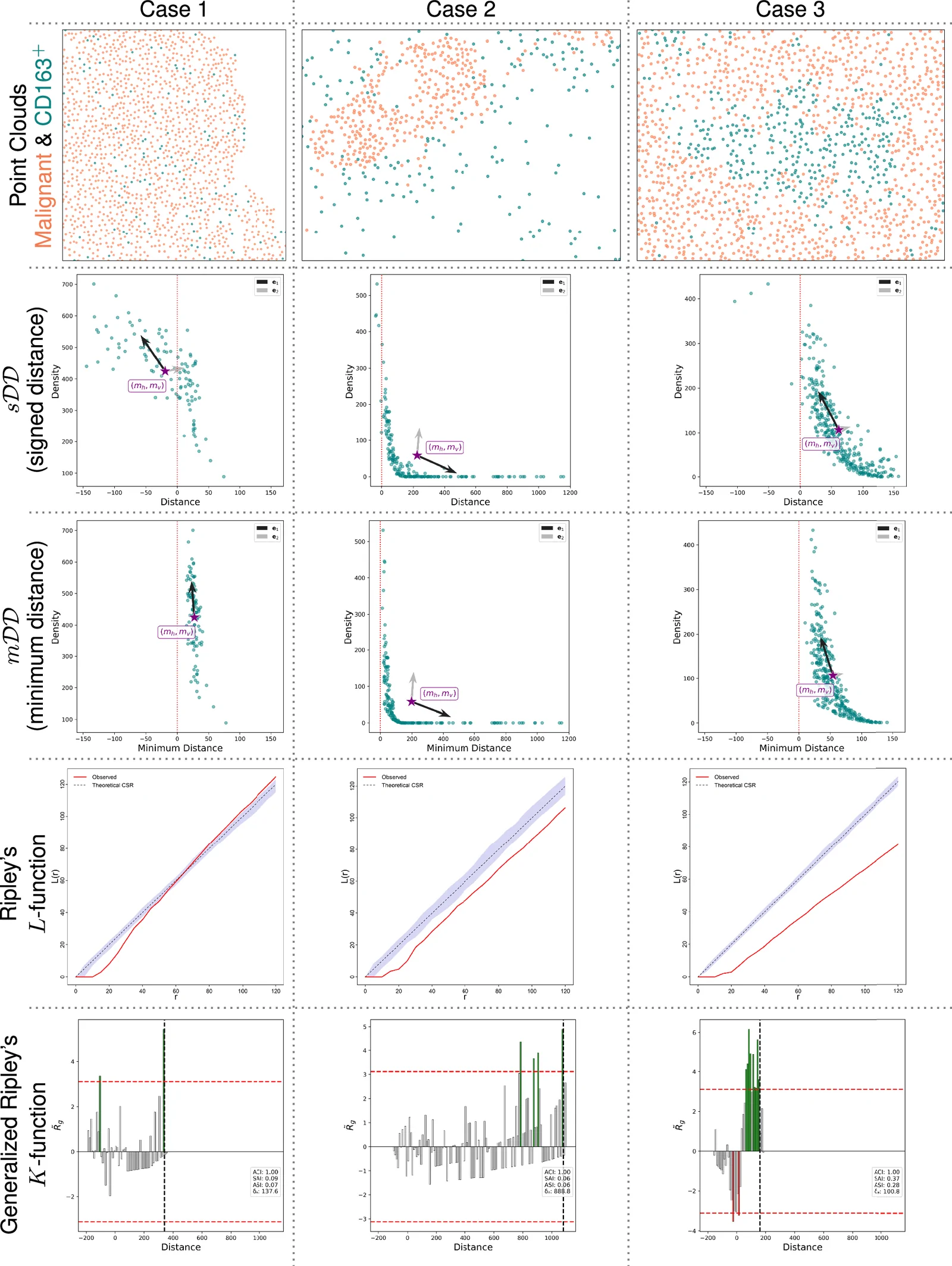
Comparison of three spatial patterns. Each column represents the three cases in the subsection $s{\mathcal{DD}}$ features distinguish intermixed, segregated and partially mixed spatial patterns: intermixed (first column), segregated (second), and partially mixed (third). The first row shows point clouds (malignant cells, coral; CD163^+^ macrophages, teal). The second and third rows display distributions in the $s{\mathcal{DD}}$ and $m{\mathcal{DD}}$ spaces with **e**_1_ and **e**_2_. The fourth row presents Ripley’s *L*-function (observed, red; CSR, black dashed; confidence interval, blue). The fifth row shows the generalized Ripley’s *K*-function across 129 distance zones (10 μm width) with statistical indices (ACI, SAI, ASI, *δ*_*a*_); green and red bars indicate cell accumulation and depletion, respectively, and gray bars denote statistically insignificant values.

We evaluate the proposed signed distance-density metric ($s{\mathcal{DD}}$) against minimum distance-density ($m{\mathcal{DD}}$), Ripley’s *L*-function, and a generalized Ripley’s *K*-function. We assess these methods across three spatial distribution patterns (Fig. 5). In Case 1 (Intermixed), immune cells are distributed throughout the malignant cell population. In Case 2 (Segregated), immune cells remain spatially separated from the malignant cell distribution. In Case 3 (Partially mixed), immune cells show partial intermingling with malignant cells while maintaining some regional separation. Mathematical expressions for these methods and additional comparisons using Ripley’s *K*- or *G*-functions, as well as our TopMR-based distance and density functions, are provided by Supplementary Figs. S1, S2 in Section S2 of Supplementary Information.

#### Proposed $s{\mathcal{DD}}$ space

In Case 1, the $s{\mathcal{DD}}$ space (first panel in the second row of Fig. 5) exhibits a linear pattern, indicating strong malignant-immune interaction. The average signed distance (*m*_*h*_ < 0), that is, the negative value of *m*_*h*_ explains that most immune cells are located within TopMR, while the high *m*_*v*_ value indicates that many malignant cells are present near immune cells. The principal direction **e**_1_ points to the top-left, indicating that immune cells deeply infiltrate TopMR in regions of high malignant cell density. In Case 2 (second panel in the second row), the $s{\mathcal{DD}}$ space displays an exponential-decay pattern with high *m*_*h*_ and low *m*_*v*_, indicating weak spatial interaction. Case 3 (third panel in the second row) shows that most immune cells (CD163^+^) are located in the central region with few malignant cells. However, along the boundaries of this central region, malignant cells are interspersed with CD163^+^ cells. This heterogeneous pattern is captured in the $s{\mathcal{DD}}$ space, where the distribution exhibits a pattern intermediate between the linear pattern in Case 1 and the exponential-decay in Case 2. The positive *m*_*h*_ value indicates that most immune cells are located outside TopMR, consistent with the central clustering of CD163^+^ cells. However, the top-left orientation of **e**_1_ indicates that some immune cells deeply infiltrate TopMR and interact with malignant cells in high-density regions. The mentioned three spatial distribution patterns can be clearly distinguishable by reading the sign of *m*_*h*_ and the direction of **e**_1_; see Supplementary Table S1 in Supplementary Section S3 of Supplementary Information.

#### $m{\mathcal{DD}}$ space

For direct comparison, we construct a minimum distance-density space ($m{\mathcal{DD}}$), which uses the same density calculation as $s{\mathcal{DD}}$ but replaces signed distance with minimum distance from each CD163^+^ cell to the nearest malignant cell. In Case 1, the $m{\mathcal{DD}}$ space (first panel in the third row) shows a linear pattern similar to $s{\mathcal{DD}}$, with an average minimum distance, *m*_*h*_ = 27.31 (see Supplementary Table S1 of Supplementary Information). In Case 2 (second panel in the third row), $m{\mathcal{DD}}$ displays an exponential-decay pattern, similar to the proposed method. In Case 3 (third panel in the third row), $m{\mathcal{DD}}$ shows an intermediate pattern with *m*_*h*_ = 54.45. However, $m{\mathcal{DD}}$ does not indicate whether immune cells are positioned inside or outside the malignant cell region. Since $m{\mathcal{DD}}$ lacks this information, similar $m{\mathcal{DD}}$ patterns can arise from either slightly shifted cell-to-cell distances or fundamentally different spatial organizations, making it difficult to determine whether Cases 1 and 3 represent similar spatial patterns with different cell-to-cell distances or qualitatively distinct organizations.

#### Ripley’s *L*-function

In Case 1, Ripley’s *L*-function (first panel in the fourth row) exceeds the theoretical complete spatial randomness (CSR) line at larger radii (approximately *r* > 60), suggesting global clustering or attraction, consistent with previous findings^8^. In Case 2, the *L*-function remains below the CSR line, indicating spatial separation between the two populations. In Case 3, the *L*-function lies far below the CSR line, likely due to local spatial isolation of the two cell types. Since this method relies on global spatial statistics and assumes homogeneity, it provides limited information for spatially heterogeneous or unevenly distributed patterns.

#### Generalized Ripley’s *K*-function

The generalized Ripley’s *K*-function^14^ was originally defined using only positive distances from a region. Since our framework requires both positive and negative distances from TopMR, we use our signed distance metric in this computation. The distance range for the generalized *K*-function is set using the global minimum and maximum distances observed across all three cases, so that a common range is used to account for differences in image size. In Case 1 (first panel in the fifth row), only green bars are observed across all distance zones, indicating an excess of CD163^+^ cells relative to a uniform reference (cell accumulation). In Case 2 (second panel in the fifth row), gray bars (statistically unreliable estimates) dominate at short distances because most CD163^+^ cells lie far from malignant cells, leaving too few neighboring points to compute stable statistics near the malignant region. Green bars appear only at large distances, indicating accumulation far from malignant cells; however, such long-distance accumulation is difficult to interpret biologically in terms of local malignant-immune interaction. In Case 3 (third panel in the fifth row), red bars (depletion) appear at short distances and green bars (accumulation) emerge at larger distances. This transition from depletion near the malignant region to accumulation farther away reflects the partially mixed pattern and provides more nuanced information than Ripley’s *L*-function in this example. Despite these advantages, the generalized Ripley’s *K*-function poses practical challenges for large-scale analysis. When a single global distance range is applied across images of varying size and malignant-immune separation, many images may produce numerous gray bars (unreliable estimates), which become zeros in the resulting feature matrix. This leads to highly sparse feature vectors, complicating clustering or classification. Conversely, if the distance range is adapted separately for each image, the physical meaning of the distance axis is no longer consistent across images: a given column in the feature matrix no longer corresponds to a fixed distance scale. In that case, feature values remain dense but lose direct interpretability in terms of absolute distances from the malignant region.

In summary, $s{\mathcal{DD}}$ distinguishes these three spatial patterns in an interpretable manner. The quantitative measures *m*_*h*_, *m*_*v*_, and **e**_1_ in the proposed feature matrix enable effective characterization of both the extent and directionality of immune infiltration across all three cases. By explicitly encoding signed distances from the TopMR boundary and evaluating tumor-cell density at the locations of immune cells along this distance axis, and by using TopMR as a global representation of malignant-cell regions, our method enables a quantitative categorization of the three cases that is biologically interpretable as high, low, or heterogeneous infiltration.

### Ball Mapper reveals continuous trajectories of malignant-immune interaction

Having quantified the per-image malignant-immune interaction as 18-dimensional vectors, we summarize their organization across all images using Ball Mapper, a topological method that represents a high-dimensional dataset as a graph of overlapping micro-clusters; the graph then provides the basis for our interpretation on the DLBCL feature matrix.

**Fig. 6:**
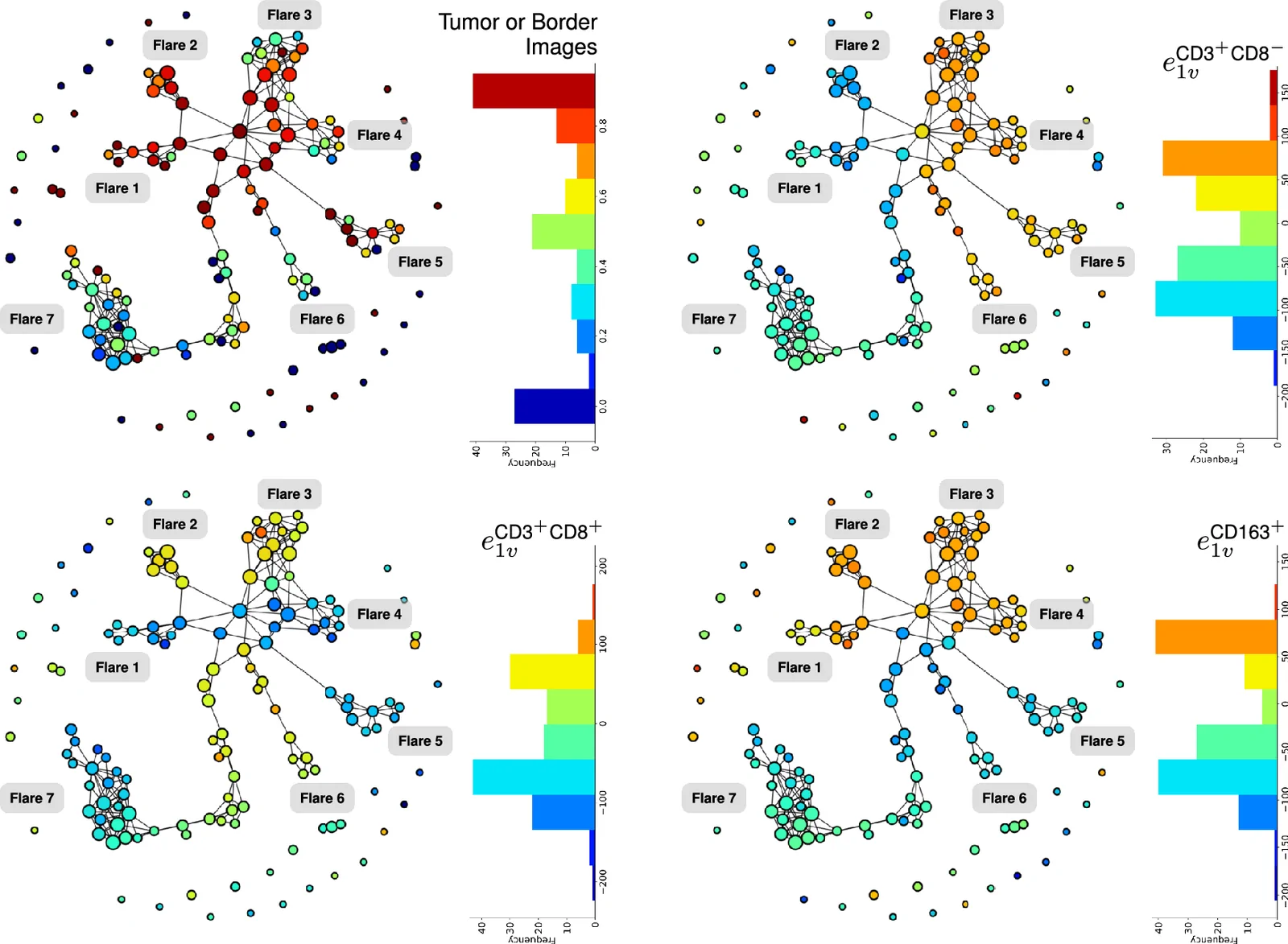
Ball Mapper of feature matrix. The top-left panel shows TR (malignant-enriched, value = 1) and BR (tumor-border, value = 0) images, with nodes colored by the mean assigned value (red = TR, blue = BR). In the 18D feature space, the graph summarizes the distribution of TR and BR images based on feature similarity (see Eq. (12)). The remaining panels visualize the vertical component of the first principal component (${{\bf{e}}}_{1v}^{i}$) for each aggregated phenotype *i* using the same color scale, with corresponding value distributions shown alongside.

The input is the feature matrix (Eq. (13)) whose rows capture immune cell infiltration and the local concentration of malignant cells across three aggregated phenotypes. Using the *cosine dissimilarity* and the parameter *ϵ* of the spatial granularity, Ball Mapper represents this dataset as a simplified graph with nodes and edges (e.g. Fig. 6). A node consists of row vectors (images) with similar characteristics; thus, each node in the graph can be regarded as a micro-cluster in the feature space. An edge between nodes indicates that there is at least one common feature shared by nodes. Adjusting *ϵ* allows control over the resolution of the graph, with smaller values producing a more detailed graph and larger values generating broader connections among features. A sensitivity analysis of the Ball Mapper graph over a ±50% range of *ϵ*_0_ = 4.5 ⋅ 10^−2^ shows that (i) *ϵ*_0_ is the value at which the dominant connected component forms, and (ii) the pairwise co-membership of samples, which samples share at least one node, is locally stable in a neighborhood of this value. Because Ball Mapper produces an overlapping cover rather than a partition, stability is assessed at the level of sample pairs rather than node identities. Full details are provided in Supplementary Section S8 of the Supplementary Information.

We now interpret the Ball Mapper graph obtained on the DLBCL feature matrix. We elaborate the meaning of the Ball Mapper graphs (Fig. 6) of the feature matrix (Eq. (13)). We label seven visually distinct branches as *Flares* only for convenience of description. The color on a node means the average of assigned scalar values on rows (images) in the node. For example, in the top-left panel, we assign value 1 to TR images and 0 to BR images; nodes are colored by the average value. Thus, a red node contains all TR images, a blue node all BR images, and intermediate colors indicate a mix of TR and BR images. Now, it is clear to see that Flares from 1 and 2 mainly consist of TR images, though not exclusively.

In the other panels of Fig. 6, we choose the color of images as ${e}_{1v}^{i}$ for $i\in {\mathcal{I}}$. Consider Flare 3 across all panels simultaneously. Examining the red nodes in Flare 3 (top-left panel), we find these nodes predominantly classified as TR images. Correspondingly, their associated panels indicate large magnitudes of ${e}_{1v}^{i}$, suggesting a nearly linear pattern in the $s{\mathcal{DD}}$ space. However, some nodes in Flare 3 with similar patterns also include BR images. This shows that both BR and TR images can share comparable patterns under the cosine dissimilarity measure. In Flare 2, nodes are mostly TR images, but ${e}_{1v}^{{{\rm{CD3}}}^{+}{{\rm{CD8}}}^{-}}$ shows a very different value than the other two immune cells. In other words, even though two images from Flares 2 and 3 are both annotated as TR, their spatial patterns in the $s{\mathcal{DD}}$ space can be different.

These observations illustrate that three typical geometric patterns in $s{\mathcal{DD}}$ space, such as the panels (vi), (vii), and (viii) in Fig. 4a or b, do not exhaustively describe all TR or BR images. The Ball Mapper graph reveals TR and BR images that deviate from, or interpolate between, these patterns, and provides a clear way to identify such intermediate cases in the high-dimensional feature space. Along each flare, node colors and feature values vary gradually, indicating that Ball Mapper organizes images along continuous trajectories of malignant-immune interaction patterns in the $s{\mathcal{DD}}$ space. This continuity arises because the summary features (Eq. (11)) used to construct the Ball Mapper graph change smoothly under moderate perturbations of the underlying $s{\mathcal{DD}}$ distributions, so small changes in spatial patterns translate into small movements along the flares.

**Fig. 7:**
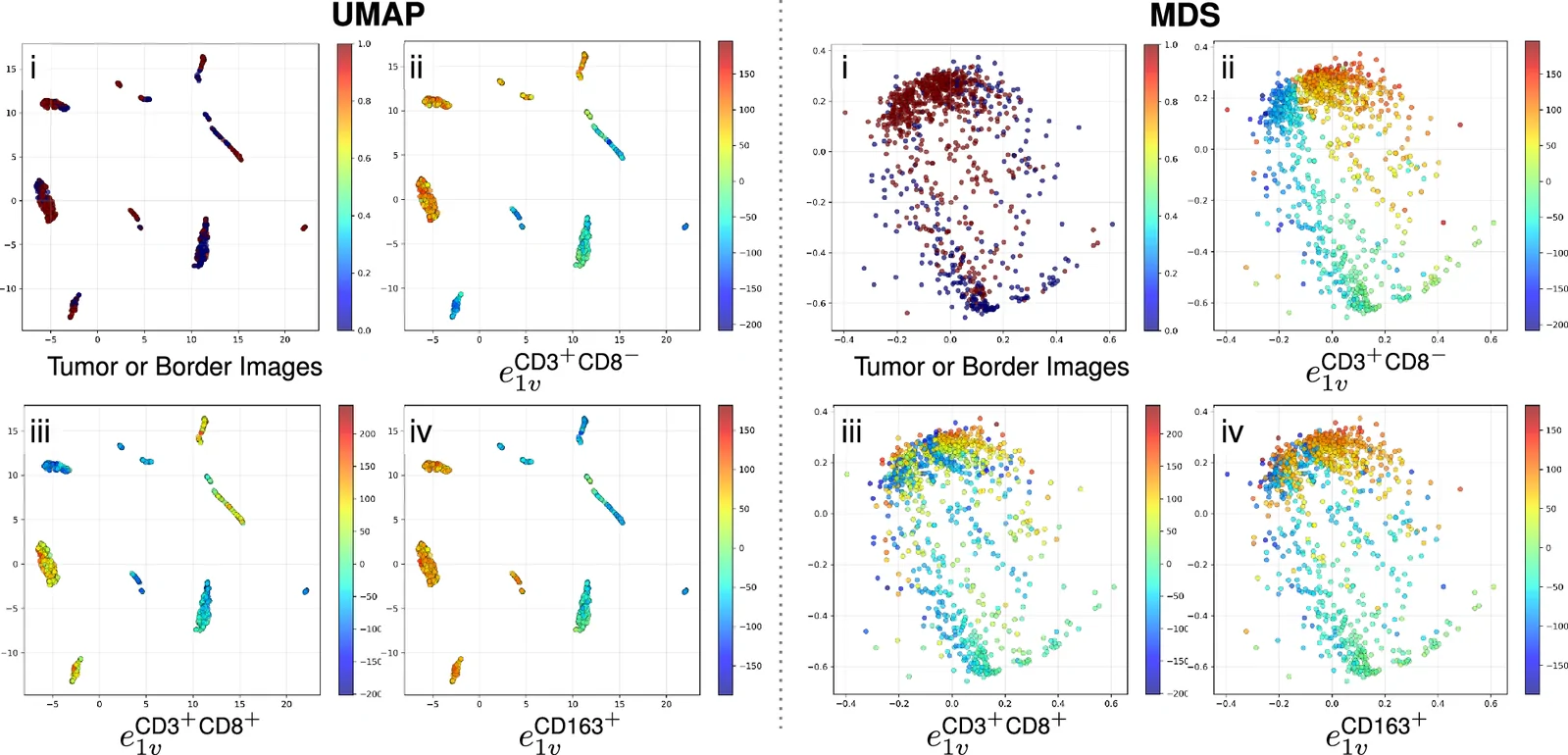
UMAP and MDS of feature matrix. The four graphs on the left and right of the dotted line are the UMAP and MDS results of feature matrix (Eq. (13)) onto 2D coordinates, respectively. For 972 points, the color of panel (i) is red = TR image and blue = BR image. We use the color of panels (ii), (iii), and (iv) is the vertical component of the first principal component (${{\bf{e}}}_{1v}^{i}$) for each aggregated phenotype *i*. UMAP fragments continuous transitions of malignant-immune cell interaction into clusters. MDS shows continuous transitions of ${e}_{1v}^{{{\rm{CD3}}}^{+}{{\rm{CD8}}}^{-}}$, but it is difficult to distinguish the information on panels (iii) and (iv).

We also examine dimensionality reduction methods, including Uniform Manifold Approximation and Projection (UMAP)^26^ and Multidimensional Scaling (MDS)^27^, applied to the same feature matrix (Eq. (13)). (See also results of t-SNE^28^ and PCA on Supplementary Fig. S3 in Supplementary Section S4 of Supplementary Information.) As shown in Figs. 7, UMAP shows visually separated clusters with some consistent properties in ${e}_{1v}^{{{\rm{CD3}}}^{+}{{\rm{CD8}}}^{-}}$, ${e}_{1v}^{{{\rm{CD3}}}^{+}{{\rm{CD8}}}^{+}}$, and ${e}_{1v}^{{{\rm{CD163}}}^{+}}$ across clusters. However, tumor and border images remain mixed within all clusters. While UMAP’s compact clustering is generally advantageous for clustering tasks, the discrete, isolated nature of these clusters makes it difficult to analyze the continuous change of spatial patterns between clusters. Consequently, this approach can limit to analyze information about the heterogeneity within and between tumor and border images, thereby restricting the ability to address the spatial diversity inherent in our data. In the MDS results, points in the reduced space appear to follow gradual transitions for ${e}_{1v}^{{{\rm{CD3}}}^{+}{{\rm{CD8}}}^{-}}$, ${e}_{1v}^{{{\rm{CD3}}}^{+}{{\rm{CD8}}}^{+}}$, and ${e}_{1v}^{{{\rm{CD163}}}^{+}}$. However, tumor and border images are quite mixed in the reduced space. Moreover, many images concentrate in the upper region, forming a large aggregation that obscures finer distinctions between images with different spatial patterns.

In contrast, the Ball Mapper is not a dimensionality reduction method and thus avoids the information loss inherent in mapping high-dimensional data into a low-dimensional space. The Ball Mapper graph preserves local connectivity directly in the original feature space, capturing continuous transitions in spatial interaction patterns without imposing artificial separations. This topologically faithful representation aligns naturally with the continuous heterogeneity of malignant-immune cell interactions and provides a more interpretable foundation for subsequent survival-linked analysis.

### Spatial interaction patterns in $s{\mathcal{DD}}$ space associate with patient survival

In this section, we assess whether the spatial interaction patterns identified above associate with patient survival outcomes. While Ball Mapper effectively captures similarity among 18*D* feature vectors from images, it operates at the image-level rather than the patient-level. Given the variable number of images per patient (ranging from 3 to 48), directly associating Ball Mapper nodes with survival outcomes (e.g., by averaging survival times across images in each node) would be biased. To address this, we adopt a two-stage hierarchical clustering (HC) strategy, using Euclidean distance with Ward linkage. Stage 1: we cluster images by their spatial interaction patterns in nodes of the Ball Mapper graph. Stage 2: For each patient, we compute the normalized distribution of their images across the clusters obtained from Stage 1. We then group patients by how their images distribute across clusters, i.e., patients with similar image distributions are grouped together. The two-stage HC yields patient-level clusters that can be used for Kaplan–Meier (KM) survival analysis.

We therefore require an algorithm for choosing cluster numbers at both stages and for providing statistical stability. To more fully explore the feature geometry in the 18*D* space and assess stability, we rerun Ball Mapper multiple times after randomly permuting the rows of the feature matrix (Eq. (13)). These permutations leave the data unchanged but produce complementary coverings, allowing us to observe the same structure in high-dimensional space from multiple views and assess which patterns are stable. With the multiple runs with permuted feature matrices, we determine the optimal number of clusters for both stages based on *p*-values from survival analysis rather than selecting them manually. Using this approach, we can both strengthen the statistical reliability of Ball Mapper and inform the optimal number of clusters for HC, thus combining the strengths of topological and statistical methods^29^.

To perform this analysis, we generate ∣*J*∣ = 1000 shuffled versions of the feature matrix by randomly permuting its row order using a uniform distribution. Let *J* denote the index set of these shuffled matrices. For each pair (*K*, *L*) of positive integers representing candidate cluster numbers, we apply the following procedure to each shuffled matrix indexed by *j* ∈ *J*:

1. Graph construction: Apply Ball Mapper with fixed *ϵ*_0_ to the *j*th feature matrix, yielding a graph with *N*_*j*_ nodes.
2. Node-level averaged features: For each node, we compute the average of the 18*D* feature vectors in Eq. (12) assigned to it, resulting in an 18 × *N*_*j*_ matrix.
3. Stage 1 image-level clustering: We apply hierarchical clustering with *K* clusters to the columns of the 18 × *N*_*j*_ matrix.
4. Stage 2 patient-level clustering: For each patient, we compute the normalized count of their images in each of the *K* clusters, yielding a *K* × *Q* matrix, where *Q* is the number of patients. We then apply hierarchical clustering with *L* clusters to the columns of this matrix.
5. Survival analysis: We compute KM survival analysis for the *L* patient clusters and *p*-value (log-rank test) *p*_*j*_(*K*, *L*).

From the steps above, in order to achieve a statistically meaningful analysis, we count the number of matrices whose KM analysis yields a *p*-value *p*_*j*_(*K*, *L*)≤*δ*(= 0.05):

14 $$\begin{array}{l}P(K,L)=\left|{J}^{{\prime} }\right|\le | J| ,\,\,\,\,{J}^{{\prime} }=\left\{j\in J| {p}_{j}(K,L)\le \delta \right\}.\end{array}$$

For reliable interpretation of survival-related features, we consider only patient clusters containing at least 10 patients, referring to these as *valid clusters*. For each matrix *j* ∈ *J*, we count the total number of patients belonging to these valid clusters, denoted by *s*_*j*_(*K*, *L*). We then sum these patient counts across all matrices, $S(K,L)={\sum }_{{j}^{{\prime} }\in {J}^{{\prime} }}{s}_{{j}^{{\prime} }}(K,L)$ (see the Supplementary Algorithm 1 in Supplementary Section S1 of the Supplementary Information).

Finally, we determine the optimal numbers of clusters K^*^ and L^*^ by maximizing the statistical significance and robustness of patient grouping as follows:

15 $$({K}^{* },{L}^{* })=\mathop{\arg \,\max }\limits_{(K,L)\in {\mathcal{K}}\times {\mathcal{L}}}\left\{P(K,L):\frac{S(K,L)}{P(K,L)}\ge \frac{T}{2}\right\},$$

where ${\mathcal{K}}=\{5,6,\ldots ,25\}$, ${\mathcal{L}}=\{3,4,\ldots ,25\}$, *T* is the total number of patients. In two-stage HC, (K^*^, L^*^) identifies the most statistically robust clustering arrangement that includes at least half of the patients.

We integrate spatial interaction patterns with survival analysis to investigate their association with patient outcomes. For our data, the total number of patients is *T* = 78 in Eq. (15), with optimal cluster numbers (K^*^, L^*^) = (8, 12).

**Fig. 8:**
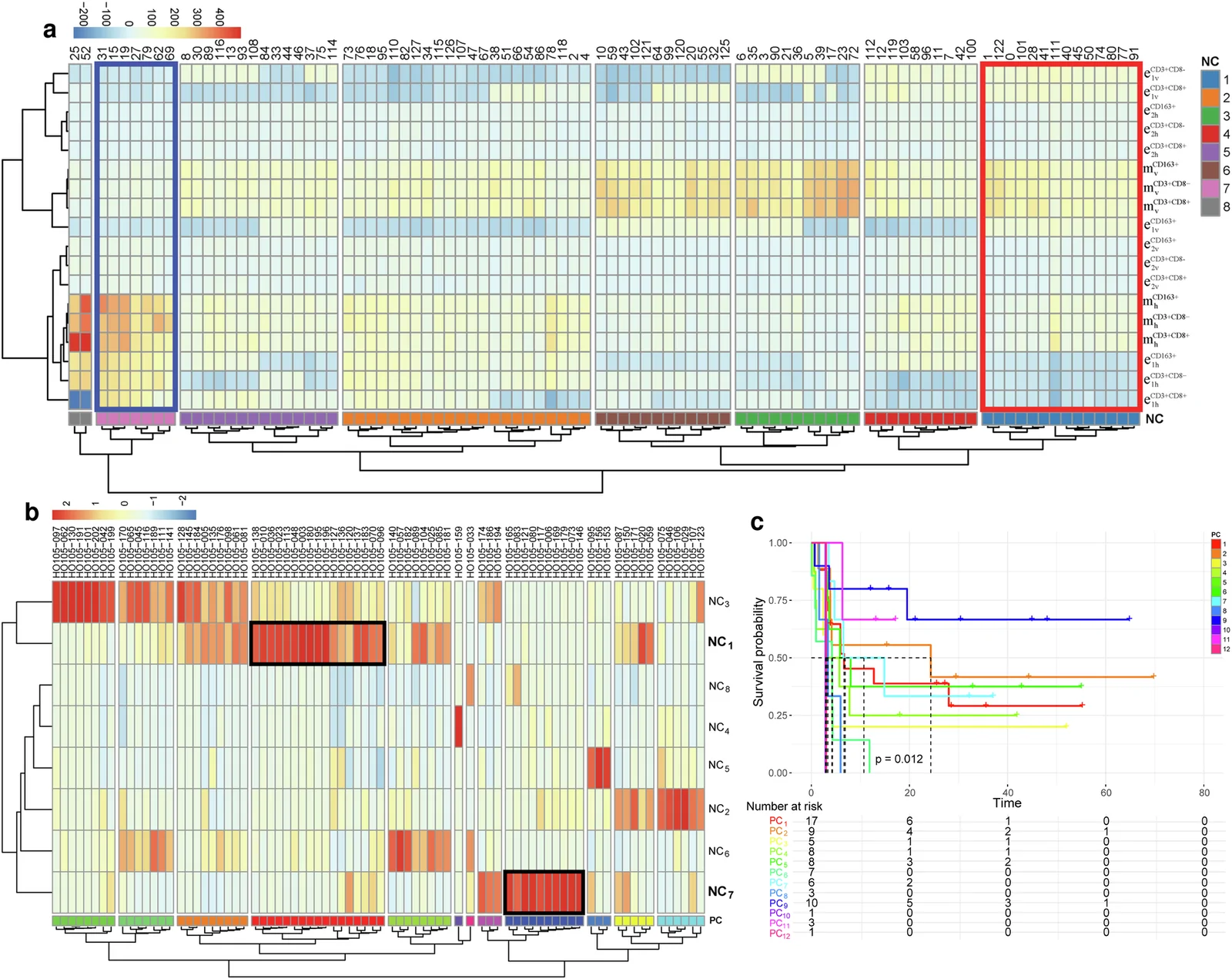
Clustering of node features and patient groups with survival analysis. **a** Heatmap generated from the feature by node matrix, showing the average values of each feature (rows) across the node numbers (columns). Hierarchical clustering is performed using Euclidean distance with the 8 clusters, named *NC* by different colors. **b** Heatmap of the *NC* frequency by patient matrix, column-scaled and clustered using Euclidean distance-based hierarchical clustering, showing the average *NC* frequency (rows) for each patient (columns). The patient clusters (*PC*s) are represented by different colors. **c** Kaplan–Meier survival analysis for each *PC*.

Figure 8 shows a two-stage HC result from the *j*^th^ shuffled feature matrix with *p*_*j*_(K^*^, L^*^)≤*δ*. Stage 1 identifies 8 Node Clusters (*NC*s), differentiated by averaged 18*D* features on nodes of Ball Mapper graph (Fig. 8a). Stage 2 distinguishes these into 12 Patient Clusters (*PC*s) (Fig. 8b), with survival probabilities shown in Fig. 8c. Among these *PC*s, *PC*_1_ and *PC*_9_ (containing ≥10 patients) are selected as valid patient clusters. Their node distributions (Fig. 8b) show *PC*_1_ predominantly linked to *NC*_1_ and *PC*_9_ to *NC*_7_, where these *NC*s exhibit the highest average composition among all *NC*s for patients within each *PC*. We designate these as dominant *NC*s for each patient cluster. Since each *NC* comprises multiple Ball Mapper nodes that track corresponding point cloud data, we computed and averaged spatial features (mean distance, density, principal components) for all phenotypes across these nodes. These averaged features and BR ratios (the proportion of border-annotated images for each patient in valid *PC*s) are then linked to survival probabilities from KM analysis. We repeat this procedure for all shuffled feature matrices satisfying *p*_*j*_(K^*^, L^*^)≤*δ* and aggregate the results to examine relationships between spatial features and survival outcomes (detailed workflow (Supplementary Fig. S4) in Supplementary Section S5 of Supplementary Information).

**Fig. 9:**
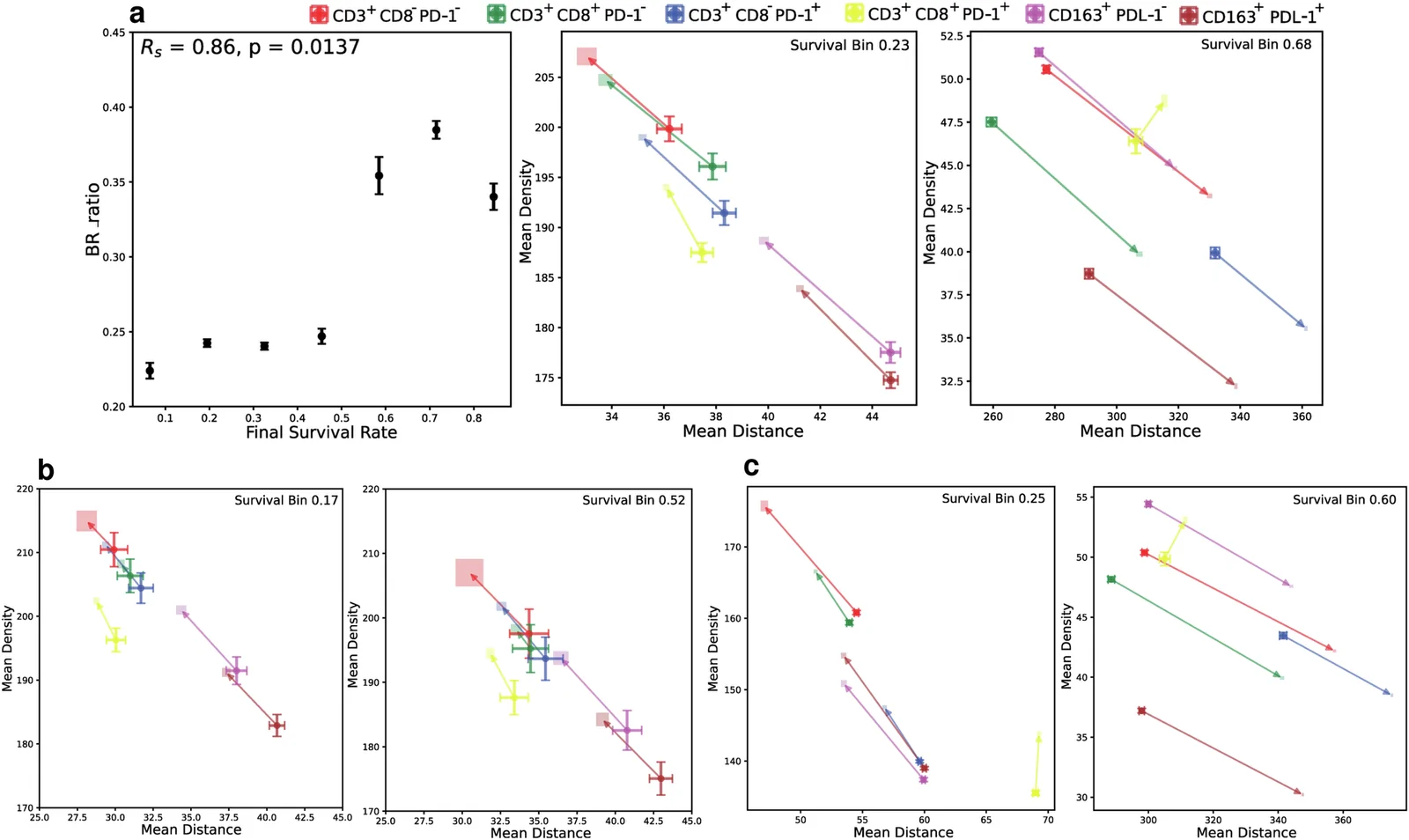
Survival analysis using TR (malignant-enriched) and/or BR (tumor-border) images and spatial features. Each panel presents results from shuffled feature matrices in different image-type conditions: TR+BR (**a**), TR only (**b**), and BR only (**c**). **a** Using 628 shuffled matrices with TR and BR combined: Left: The relationship between final survival rate and the border-image ratio for corresponding patients (BR_ratio_). The border-image ratio is averaged within each bin of the final survival rate, using a bin size of 0.13. The Spearman’s Rank Correlation Coefficient (*R*_*s*_) of 0.86 indicates a strong positive monotonic relationship. Middle & Right: Mean distance, mean density, and mean **e**_1_ across all phenotypes for the poor survival group (median survival probability = 0.23) and the good survival group (=0.68), respectively. Standard errors for distance and density are shown for each phenotype, while mean **e**_1_ is represented by arrows with error ranges (rectangles). For better visualization, we scale **e**_1_ by multiplying it by 0.05. **b** TR only; based on 184 shuffled matrices, median survival probabilities: 0.17 (poor group) and 0.52 (good group). **c** BR only; based on 784 shuffled matrices, median survival probability: 0.25 (poor group) and 0.60 (good group).

Based on the aggregated results, we divided patients into two survival groups and computed the mean values of spatial interaction features (*m*_*h*_, *m*_*v*_, *e*_1*h*_, and *e*_1*v*_) of each group to identify trends linking spatial interaction to survival outcomes. These results are visualized in a *mean*$s{\mathcal{DD}}$ space (Fig. 9), integrating all phenotypes to provide a comprehensive view of malignant-immune interactions. For visualization, we excluded outliers beyond the interquartile range, which reduced the skewness of nearly all features to below 0.5.

Figure 9a (left) shows a positive correlation between the proportion of BR images and survival probability, indicating that *PC*s with higher survival probability have more BR images. We interpret that tumors with more visible borders (not a sampling artifact, since TR/BR selection is independent of outcome) may be linked to improved survival outcomes (see Discussion). Figure 9a (middle, right) compares poor vs. good survival groups in mean $s{\mathcal{DD}}$. The good survival group shows significantly lower mean malignant density for all phenotypes, indicating fewer malignant cells nearby. Additionally, the mean **e**_1_ orientation (bottom-right) in the right panel suggests that patients in the good survival group have a higher proportion of BR images, consistent with the BR pattern in Fig. 4b. Since BR and TR images exhibit distinct distance and density scales, analyzing them together may obscure interpretation due to scale mixing. This motivates separate analysis of TR and BR images to accurately characterize spatial interaction patterns. We therefore applied the two-stage HC separately to TR (*T* = 74) and BR (*T* = 54) images in Eq. (15). The optimal number of clusters were (K^*^, L^*^) = (7, 13) for TR and (K^*^, L^*^) = (6, 8) for BR.

Figure 9b (TR only) shows that all phenotypes in the poor survival group present a higher mean malignant cell density than in the good survival group. In the good survival group, CD8^+^ T cell subsets (CD3^+^CD8^+^PD-1^−^ and CD3^+^CD8^+^PD-1^+^) are positioned closer to the strong infiltration zone of the mean $s{\mathcal{DD}}$ space than other phenotypes, the pattern not observed in the poor survival group. This pattern may indicate that CD8^+^ T cell subsets interact more actively with malignant cells in the good survival group. Furthermore, in the good survival group, CD3^+^CD8^−^PD-1^−^ cells have a much larger mean **e**_1_ magnitude than in the poor survival group, indicating a more dispersed distribution in the original $s{\mathcal{DD}}$ space (they are more widespread in the malignant-enriched regions). Overall, all phenotypes in the good survival group are located closer to each other in the mean $s{\mathcal{DD}}$ space.

When considering BR images only, the mean $s{\mathcal{DD}}$ patterns differ noticeably between the good and poor survival groups (Fig. 9c). The poor survival group for BR images exhibits mean $s{\mathcal{DD}}$ patterns similar to those for TR images (Fig. 9b). This suggests that spatial interaction patterns of BR images may have considerable influence on patient survival outcomes.

## Discussion

This study presents a computational framework based on TDA that enables end-to-end analysis (Supplementary Fig. S5 in Supplementary Section S6 of Supplementary Information) of malignant-immune spatial interactions, from interpretable feature extraction (considering both global and local aspects) to patient survival interpretation. We first computationally define regions of malignant cells, called TopMR, based on the topological persistence of the distribution. At the global scale, immune cell infiltration is quantified by signed distance functions from TopMR boundaries, which are robust against noise (e.g., cell-center extraction errors). Locally, malignant cell densities around individual immune cells are assessed using density functions. PCA summarizes each image’s point cloud data in the signed distance-density ($s{\mathcal{DD}}$) space to characterize malignant-immune interactions. Key features (averaged signed distance, averaged density, and the first two principal components) are visualized using Ball Mapper graphs. These graphs reveal continuous transitions of spatial interaction patterns linked to patient survival outcomes. To ensure robustness, we repeatedly apply Ball Mapper to shuffled feature matrices, creating multiple perspectives on the data structure. Each Ball Mapper graph is further summarized into clusters via a two-stage HC. To interpret the relationship between spatial interactions and survival, we associate final survival probabilities (KM analysis) with the averaged spatial features (Ball Mapper nodes) across immune phenotypes. From these associations, we construct a representative *mean*$s{\mathcal{DD}}$ space by dividing cases into two survival groups. We use this averaged space, excluding outliers, to highlight general trends in malignant-immune interactions while mitigating artifacts from small sample sizes or phenotype mislabeling.

This framework provides two key advantages for spatial TME analysis. First, the automatic extraction of regions of malignant cells (TopMR) based on topological persistence provides an objective reference for quantifying immune-malignant spatial relationships. The spatial positioning of immune cells (whether infiltrating, at boundaries, or excluded) can be associated with immunotherapy response and patient prognosis, however manual tumor boundary annotation can vary between pathologists, particularly when boundaries are ambiguous. Second, the integration of TDA and statistical methods enables comprehensive end-to-end analysis linking spatial patterns to clinical outcomes. By capturing malignant-immune interactions at both global and local scales and connecting these features to survival through interpretable visualization and clustering, our approach provides a complete pipeline from computational spatial analysis to patients’ survival interpretation.

We apply the framework to DLBCL data, where our approach robustly handles complex and heterogeneous spatial cell distributions from both TR and BR images. The separate annotation of TR and BR images given by pathologists is expected to be important because the malignant-enriched regions or border regions of the tumors often represent distinct biological contexts with different immune infiltration patterns. In the patient cohort used in this study (see also Supplementary Table S2 in Supplementary Section S7 of Supplementary Information), our analysis revealed distinct spatial patterns in the mean $s{\mathcal{DD}}$ space associated with event-free survival probabilities between TR and BR regions. Higher proportions of BR images correlate with improved survival, suggesting that well-defined tumor borders are beneficial clinically. This may relate to a previous report^30^ where a diffusely infiltrative PCNSL subtype (lymphomatosis cerebri) was difficult to diagnose and associated with poor prognosis. This suggests that ill-defined, widespread tumor infiltration may correlate with worse outcomes. However, further biological and molecular studies are needed to clarify any causal relationship.

In TR images, a notable survival-related difference is the positioning of CD8^+^ T cell subsets (CD3^+^CD8^+^PD-1^−^ and CD3^+^CD8^+^PD-1^+^) in the mean $s{\mathcal{DD}}$ space. In patients with good survival, these T cells are positioned closer to the zone of strong immune infiltration than other phenotypes, a feature absent in the poor survival group. These T cells are located near the TopMR periphery with reduced malignant cell density in their vicinity, possibly reflecting active tumor clearance by CD8^+^ T cells in the good survival group. Beyond CD8^+^ T cell positioning, the good survival group shows closer spatial proximity among all immune phenotypes in the mean $s{\mathcal{DD}}$ space compared to the poor survival group. This closer positioning may indicate more coordinated immune activity or enhanced immune surveillance, though the underlying mechanisms require further investigation. In addition, CD3^+^CD8^+^PD-1^−^ cells show a larger mean **e**_1_ magnitude, indicating widespread distribution in the original $s{\mathcal{DD}}$ space. This dispersed pattern is associated with survival advantages, likely enhancing immune efficacy.

Analyzing BR images independently, we find that patients whose tumor border regions exhibit spatial TME patterns resembling those of malignant-enriched regions (characterized by high malignant cell density and infiltration) have lower survival probabilities. This pattern suggests that infiltrative tumor borders may represent an adverse prognostic indicator in DLBCL, similar to findings in colorectal cancer^31^ and pancreatic neuroendocrine tumors^32,33^.

Despite the strengths of the proposed framework, several aspects warrant further development. The PCA-based pattern characterization in the $s{\mathcal{DD}}$ space offers parameter-free, compact features that respond continuously to variations within the normal range, but it can be sensitive to large variations. We apply the framework to a DLBCL cohort of 78 patients. The reported survival associations are statistically significant within this cohort and may have prognostic relevance. However, these associations are not adjusted for established clinical or molecular prognostic factors and remain to be validated in an independent cohort. Larger cohorts that account for these factors are needed to establish their generalizability and prognostic value. Broader clinical translation also faces a practical barrier, as the mIF imaging used in this study is more resource-intensive than routinely available whole-slide imaging^34^, motivating future adaptation of the framework to more accessible imaging modalities. Although the spatial patterns of PD-1^+^ and PD-L1^+^ cells can be examined by tracing back to individual patients within clusters, the aggregated nature of the clustering approach limits detailed interpretation specific to these activation markers. Potential future directions include enhancing robustness against such variability, expanding the application to multiple cohorts and cancer types, and developing a classifier that fully utilizes spatial interaction features for clinical outcome prediction. Additionally, while this study focuses on malignant-immune interactions, exploring spatial interactions among different immune cell types represents a valuable direction for extending this framework. Since our framework operates on pre-processed point data instead of raw images, it is not tied to a specific imaging platform or preprocessing pipeline. Applying it to other spatial data types that provide cell-level coordinates and phenotype annotations, such as single-cell spatial transcriptomics platforms (e.g., Xenium and CosMx), represents another promising direction.

## Methods

### Data acquisition and pathologist annotation

We use the patient data and point clouds from mIF images (e.g. Fig. 1) of primary central nervous system DLBCL patients and the image segmentation and phenotype classification are performed using InForm software (Akoya Biosciences) as described in refs. ^8,35^. The images were independently annotated by pathologists as two types, ensuring no patient-specific bias:

- TR image: the malignant-enriched regions of the tumors.
- BR image: the border regions of the tumors.

Pathologists selected these regions based on qualitative criteria such as malignant cell infiltration; TR images have high malignant cell concentrations, while BR images show tumor interactions with adjacent tissue.

From these annotated images, we extract the locations of malignant cells (PAX5^+^) as the reference point cloud ${\mathcal{R}}$ and the locations of three aggregated immune cell types as ${{\mathcal{Q}}}^{i}$ (Eq. (3)). The formal mathematical definitions of these point clouds and the index set are stated at the start of the Results section (Eqs. (1)–(3)). We aggregate immune cell phenotypes to maximize image inclusion: CD3^+^CD8^−^, CD3^+^CD8^+^, and CD163^+^; colors in Table 1. This aggregation is necessary because not every image contains all phenotypes, and PD-1^+^ and PD-L1^+^ cells less frequently observed in this dataset.

**Table 1:** Legend of cell phenotypes and corresponding colors

	

### Persistent homology and simplicial complex construction

The persistent homology computation described in the Results section is performed using the GUDHI library^21^. Pairwise Euclidean distances among malignant cell positions are precomputed, and a Vietoris–Rips complex is constructed with the simplex tree built up to dimension two. The filled riangles of the extracted simplicial complex are rasterized into a binary mask, which serves as the input for the subsequent signed distance computation.

### Signed distance field and density estimation

The signed distance field *φ*(**x**) defined in Eq. (7) is computed numerically by solving the eikonal equation^25^ via the Fast Marching Method, as implemented in the scikit-fmm Python library. Using a discrete grid domain, $\widetilde{\Omega }=\{(l,k)\in \Omega :l,k\in {\mathbb{N}}\}$ (${\mathbb{N}}$: set of natural numbers), let *M* denote the binary mask on $\widetilde{\Omega }$, where *M*(*l*, *k*) = 1 for grid points inside TopMR and *M*(*l*, *k*) = 0 otherwise. The boundary of the mask is defined as

16 $$\begin{array}{l}\partial {\mathcal{T}}=\{(l,k)\in \widetilde{\Omega }:M(l,k)=1\,\,\mathrm{and}\,\,\exists \,({l}^{{\prime} },{k}^{{\prime} })\in {{\mathcal{N}}}_{(l,k)}\,\,\mathrm{such\; that}\,\,M({l}^{{\prime} },{k}^{{\prime} })=0\},\end{array}$$

where ${{\mathcal{N}}}_{(l,k)}=\{(l\pm 1,k),(l,k\pm 1)\}\cap \widetilde{\Omega }$ denotes the four index neighbors in $\widetilde{\Omega }$. The Fast Marching Method propagates distances outward from $\partial {\mathcal{T}}$, and the sign is assigned according to Eq. (7). The density field $\bar{\psi }({\bf{x}})$ described in Eq. (8) is computed by summing Gaussian contributions from each malignant cell position and subsequently smoothing the result with a Gaussian filter (gaussian_filter from the scipy.ndimage module).

### Ball Mapper graph construction

The Ball Mapper graphs presented in the Results section are constructed using the pyballmapper Python library^20^. Given a point cloud *X* equipped with a dissimilarity measure and a radius parameter *ϵ*, Ball Mapper constructs an open cover of *X* by balls of radius *ϵ*. The algorithm iterates through the points and, for each point not yet covered, creates a ball of radius *ϵ* centered at that point; all points within the ball are assigned to the corresponding node. Because a single point can lie within multiple balls, the resulting cover is overlapping. A graph is then formed by placing an edge between any two nodes that share at least one common point. In this study, the cosine dissimilarity is used as the distance measure, and the radius *ϵ*_0_ = 4.5 ⋅ 10^−2^ is selected as described in the Results section.

### Hierarchical clustering and survival analysis

The two-stage hierarchical clustering (HC) and survival analysis described in the Results section are implemented in R. At both stages, HC is performed using the Ward.D2 linkage criterion with Euclidean distance, via the pheatmap R package; cluster assignments are obtained with the cutree function. In Stage 1, Ball Mapper nodes are clustered by their averaged 18-dimensional features to form Node Clusters. In Stage 2, patients are clustered by the normalized distribution of their images across these Node Clusters to form Patient Clusters. Kaplan–Meier survival curves are estimated using the survfit function from the survival R package, and *p*-values are obtained from the log-rank test as implemented in the survminer R package.

## Supplementary information

Supplementary information

## Data Availability

The processed multiplex immunofluorescence images analyzed in this study have been deposited at Zenodo and are publicly available (https://doi.org/10.5281/zenodo.7895745)^35^, as originally reported by Roemer et al.^8^. Any additional information required to reanalyze the data reported in this paper is available from the corresponding author upon reasonable request.

## Code availability

The code used in this study is implemented in Python and is publicly available at https://github.com/sapark1014/TopMR_sDD. Further inquiries regarding the code can be directed to Dr. Seol Ah Park.
