# Supplementary material for: Spatial analysis of malignant-immune cell interactions in the tumor microenvironment using topological data analysis
Source: NPJ Syst Biol Appl. 2026 Jun 13;12:141. doi: 10.1038/s41540-026-00763-7 (PMC13620163; doi:10.1038/s41540-026-00763-7)
Supplement: Supplementary file 1 [file 41540_2026_763_MOESM1_ESM.pdf]

Supplementary Information: Spatial Analysis of Malignant-Immune Cell Interactions in the Tumor Microenvironment Using Topological Data Analysis

Seol Ah Park[1], Davide Gurnari[2], Paweł Dłotko[3], Yongsoo Kim*[4], and Jooyoung Hahn*[5]

[1] *Department of Dosimetry and Application of Ionizing Radiation, Faculty of Nuclear Sciences and Physical Engineering, Czech Technical University in Prague*

[2] *Dioscuri Centre in Topological Data Analysis, Institute of Mathematics, Polish Academy of Sciences, Warsaw, Poland*

[3] *Discouri Center for Topological Data Analysis, Center of Trustworthy AI for Life Sciences - International Research Agendas Programme, University of Warsaw, Poland*

[4] *Amsterdam UMC, Vrije Universiteit Amsterdam, Department of Pathology, Cancer Center Amsterdam, Amsterdam, The Netherlands*

[5] *Department of Software Engineering, Faculty of Nuclear Sciences and Physical Engineering, Czech Technical University in Prague, Czech Republic*

S1 Algorithm of Optimal Cluster Identification

Algorithm 1 provides a step-by-step procedure for determining the optimal cluster numbers (K^*, L^*) via statistical assessment. The algorithm computes two key quantities for each candidate pair (K, L): $P(K, L)$, the number of permuted feature matrices yielding statistically significant survival differences (log-rank test p-value $\leq \delta$), and $S(K, L)$, the total count of patients across all valid clusters (those with at least 10 patients) summed over all matrices.

Algorithm 1

Data Preparation: Prepare $|J| = 1000$ matrices by randomizing the order of rows in the feature matrix.
for each $(K, L) \in \mathcal{K} \times \mathcal{L}$ **do**
  for each jth matrix in J **do**
    Compute Ball Mapper with the parameter ϵ.
    Average $[\mathbf{m}^i, \mathbf{e}_1^i, \mathbf{e}_2^i]$ for $\forall i \in \mathcal{I}$ on nodes.
    Denote N_j is the number of nodes.
    HC on Nodes with K clusters on $18 \times N_j$ matrix.
    Denote Q is the number of patients.
    HC on Patients with L clusters on $K \times Q$ matrix whose column vector is obtained by counting and normalizing the number of images per patient assigned to each of the K clusters.
    Use Kaplan-Meier analysis to compute p-value $p_j(K, L)$.
    $s_j(K, L)$ sums patients in clusters with ≥ 10 members.
  end for
  $P(K, L) = \left|J'\right|, \;\; J' = \{j \in J \mid p_j(K, L) \leq \delta\}$.
  $S(K, L) = \sum_{j' \in J'} s_{j'}(K, L)$.
end for
Optimal Cluster Selection:
Define the feasible set $\mathcal{F} = \left\{(K, L) \in \mathcal{K} \times \mathcal{L} : \frac{S(K,L)}{P(K,L)} \geq \frac{T}{2}\right\}$.
$(K^*, L^*) = \arg\max_{(K,L) \in \mathcal{F}} P(K, L)$.

*Corresponding Authors: jooyoung.hahn@fjfi.cvut.cz, yo.kim@amsterdamumc.nl

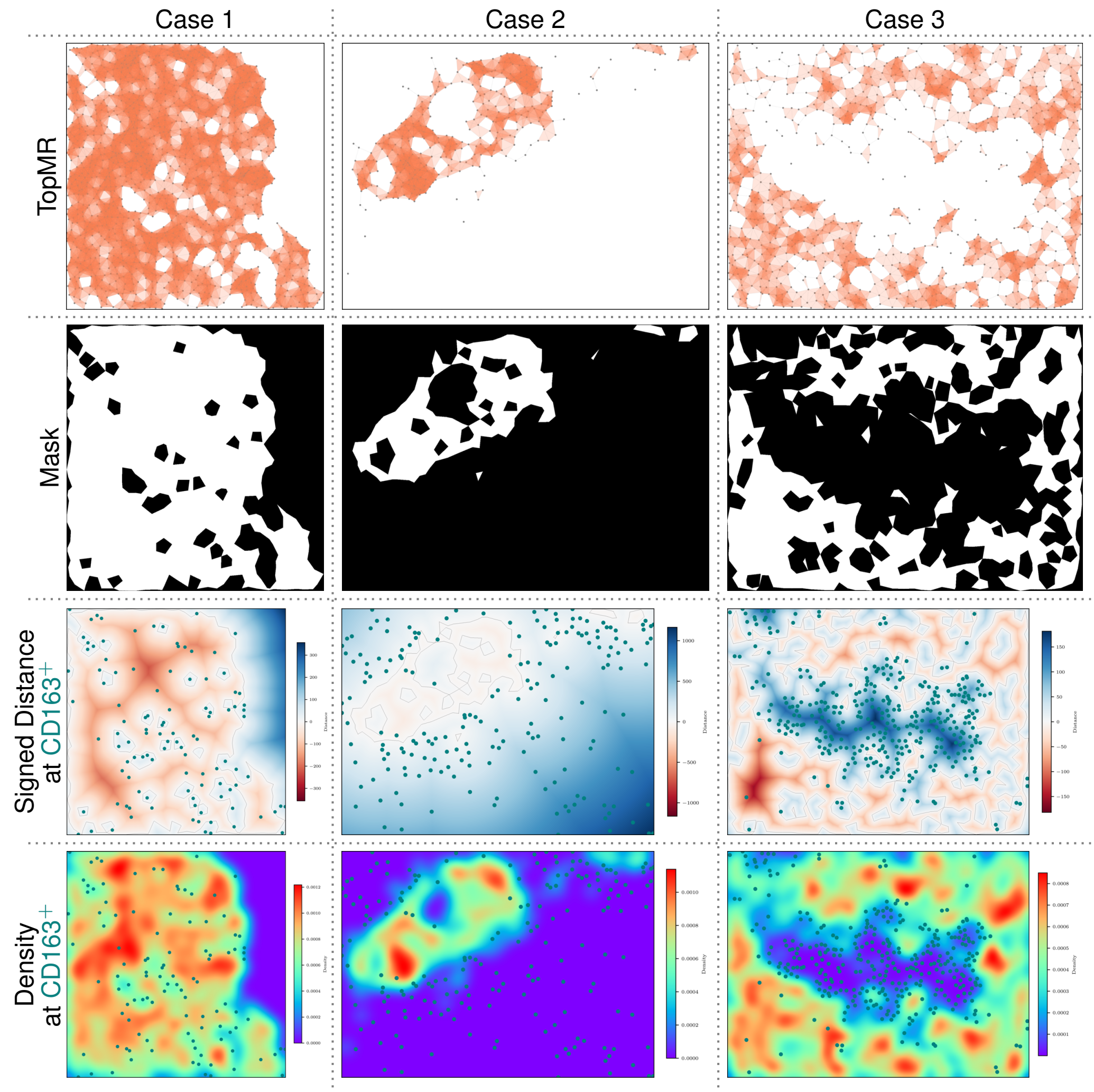

Fig. S1: Each column corresponds to three cases in the subsection *$s\mathcal{DD}$ features distinguish intermixed, segregated and partially mixed spatial patterns* of the main text: Intermixed (left), low (Segregated), and Partially mixed (right). The first row shows the computed TopMR. The second row displays binary masks, the union of TopMR triangles in white. The third and fourth rows present the signed distance and density functions, respectively, with macrophages (CD163^+) overlaid.

S2 Comparison of Spatial Metrics

For the three cases presented in the subsection *$s\mathcal{DD}$ features distinguish intermixed, segregated and partially mixed spatial patterns* of the main text, Fig. S1 shows TopMR-based distance and density functions (Case 1: Intermixed, Case 2: Segregated, and Case 3: Partially mixed). Additional comparisons using G- and Ripley's K-functions are provided in Fig. S2. Similar to the results obtained with Ripley's L-function, both G- and Ripley's K-functions distinguish Case 1 from the other cases, but show no significant difference between Case 2 and Case 3.

The mathematical formulations used for comparison are described below. For each point of the ith aggregated phenotype $\mathbf{q}^i$, the minimum distance to the location of a malignant cell ($\mathcal{R}$) is computed as

$$d_{\text{min}} = \min_{\mathbf{x} \in \mathcal{R}} |\mathbf{x} - \mathbf{q}^i|. \tag{1}$$

For Ripley's L function, from each $\mathbf{q}^i \in \mathcal{Q}^i$, the degree of spatial clustering or dispersion to malignant cells $\mathbf{x} \in \mathcal{R}$ within distance r is measured by

$$L_{\mathcal{Q}^i,\mathcal{R}}(r) = \sqrt{\frac{K_{\mathcal{Q}^i,\mathcal{R}}(r)}{\pi}}, \tag{2}$$

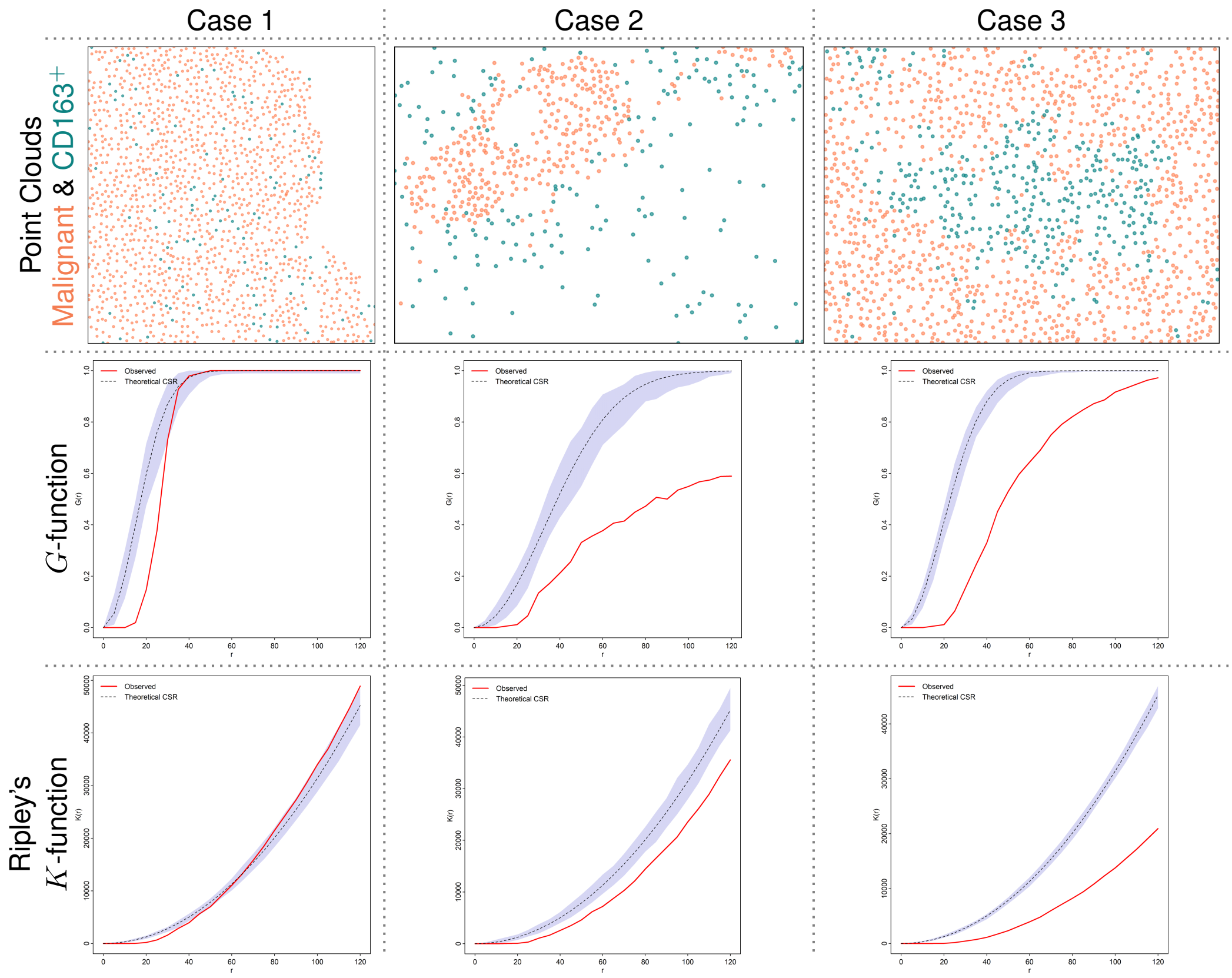

Fig. S2: Additional results to Fig. 5 in the subsection *$s\mathcal{DD}$ features distinguish intermixed, segregated and partially mixed spatial patterns* of the main text: Each row corresponds to three cases, Intermixed (top row), Segregated (middle row), and Partially mixed (bottom row). The first column displays the point cloud, where malignant cells are shown in coral and CD163$^+$ (macrophages) are shown in teal. The second and third columns present the results of G- and Ripley's K-functions, with the observed curve in red and the theoretical curve under complete spatial randomness (CSR) shown as a black dashed line, along with confidence interval shaded in blue.

Table S1: Quantitative comparison across three spatial patterns (intermixed, segregated, and partially mixed): The table summarizes key spatial features computed from the $s\mathcal{DD}$ and $m\mathcal{DD}$ spaces in Fig.5 in the main text. m_h and m_v represent the average distance and density, respectively, for each method. e_{1h} and e_{1v} denote the horizontal and vertical components of the first principal vector.

Feature	Case 1		Case 2		Case 3	
	$s\mathcal{DD}$	$m\mathcal{DD}$	$s\mathcal{DD}$	$m\mathcal{DD}$	$s\mathcal{DD}$	$m\mathcal{DD}$
m_h	−19.40	27.31	224.51	197.47	61.67	54.45
m_v	424.78	424.78	57.92	57.92	106.49	106.49
e_{1h}	−38.94	−4.57	253.62	250.81	−33.13	−19.96
e_{1v}	114.05	114.54	−48.93	−41.77	86.76	86.97

where $K_{\mathcal{Q}^i,\mathcal{R}}(r)$ is Ripley's K function, defined as

$$K_{\mathcal{Q}^i,\mathcal{R}}(r) = \frac{1}{\lambda_{\mathcal{R}}|\mathcal{Q}^i|} \sum_{\mathbf{q}^i \in \mathcal{Q}^i} \sum_{\mathbf{x} \in \mathcal{R}} I(|\mathbf{x} - \mathbf{q}^i| \leq r), \tag{3}$$

where $\lambda_{\mathcal{R}}$ is the density of malignant cells, $|\mathcal{Q}^i|$ is the number of points in the ith aggregated phenotype, and $I(\cdot)$ is the indicator function defined as

$$I(x) = \begin{cases} 1, & \text{if } x \text{ is true,} \\ 0, & \text{otherwise.} \end{cases} \tag{4}$$

The G-function is computed by

$$G_{\mathcal{Q}^i,\mathcal{R}}(r) = \frac{1}{|\mathcal{Q}^i|} \sum_{\mathbf{q} \in \mathcal{Q}_i} I\big(d_{\min}(\mathbf{q}) \leq r\big). \tag{5}$$

S3 Quantitative Comparison of $s\mathcal{DD}$ and $m\mathcal{DD}$ Methods

Table S1 quantitatively shows that $s\mathcal{DD}$ provides clearer differentiation of spatial patterns among three cases: Case 1 (Intermixed), Case 2 (Segregated), and Case 3 (Partially mixed). Based on the sign and magnitude of (m_h, e_{1h}), $s\mathcal{DD}$ (signed distance-density) clearly separates all three cases. In contrast, $m\mathcal{DD}$ (minimum distance-density) cannot clearly distinguish Case 1 from Case 3.

Based on the sign and magnitude of (m_h, e_{1h}), $s\mathcal{DD}$ (signed distance-density) clearly separates all three cases: Case 1 exhibits negative m_h with top-left $\mathbf{e}_1$ ($e_{1h} < 0$), Case 2 shows positive m_h with right-oriented e_1 ($e_{1h} > 0$), and Case 3 displays positive m_h but top-left e_1 ($e_{1h} < 0$).

S4 Limitations of t-SNE and PCA in Representing $s\mathcal{DD}$-based Spatial Patterns

This section presents results from t-distributed Stochastic Neighbor Embedding (t-SNE) and Principle Component Analysis (PCA). t-SNE provides visually separated clusters (Fig. S3), similar to UMAP (Fig. 7 in the main text). Like UMAP, the discrete and isolated clustering structure makes it difficult to track continuous variations in spatial patterns. This separation obscures gradual transitions between different spatial interaction patterns and limits the ability to capture heterogeneity across tumor and border images. Additionally, distances between clusters in t-SNE do not reflect distances in the original feature space, further limiting the ability to characterize the heterogeneous nature of the data in this study. PCA, while deterministic and globally linear, explains only a modest fraction of the variance in these $s\mathcal{DD}$-derived features. This results in substantial overlap between TR and BR images in the reduced space, making it difficult to distinguish different spatial interaction patterns.

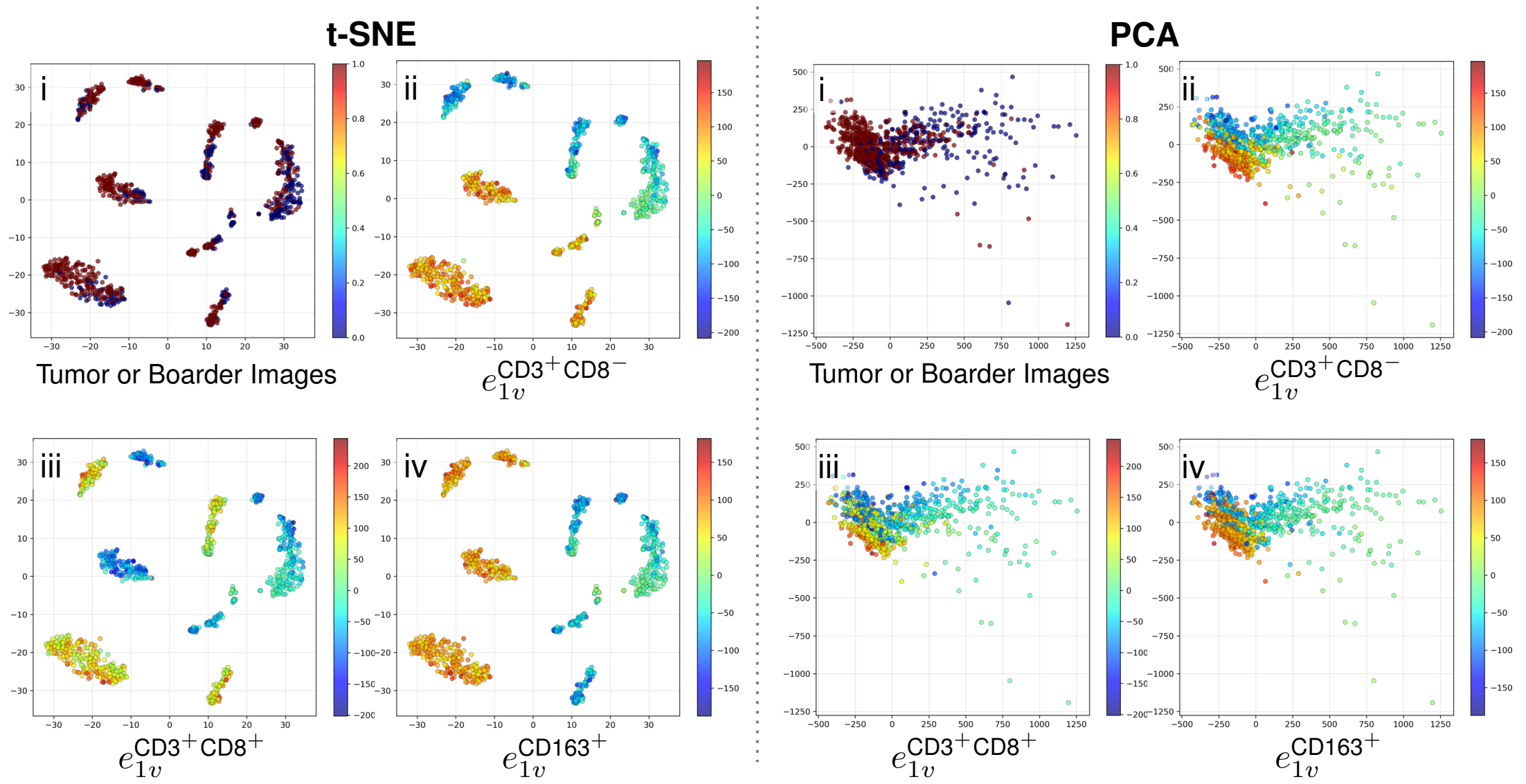

Fig. S3: The four graphs on the left and right of the dotted line are the t-SNE and PCA results of proposed feature matrix onto 2D coordinates, respectively. For 972 points, the color of panel (i) is red = TR image and blue = BR image. We use the color of panels (ii), (iii), and (iv) is the vertical component of the first principal component (e^i_{1v}) for each aggregated phenotype i.

S5 Detailed Steps for Associating Spatial Features with Survival Outcomes

Fig. S4 illustrates the workflow for linking spatial features to survival probabilities. The analysis proceeds through the following steps:

Step 1: Node Cluster (NC) Identification We perform the first stage of hierarchical clustering (1st HC) on Ball Mapper nodes based on their averaged 18D features. These features consist of six spatial metrics (mean distance, density, e_{1h}, e_{1v}, e_{2h}, e_{2v}) computed for three aggregated phenotypes.

Step 2: Patient Cluster (PC) Identification For each patient, we compute the normalized frequency distribution of their images across the node clusters from Step 1. We then apply the second stage of hierarchical clustering (2nd HC) to group patients based on these distributions. Survival probabilities for each patient cluster are computed using Kaplan-Meier analysis. We consider patient clusters containing at least 10 patients as valid clusters.

Step 3: Dominant NC Assignment For each valid PC, we identify its dominant NC based on the normalized frequency distribution of patient images across NCs, where the dominant NC exhibits the highest average composition. This reflects that the majority of images from patients in these PCs are mapped to their respective dominant NCs.

Step 4: Spatial Feature Extraction Each dominant NC contains multiple nodes in the Ball Mapper graph. Since we can track all corresponding images in each Ball Mapper node, we compute spatial features (mean distance, density, e_{1h}, e_{1v}, e_{2h}, e_{2v}) for all phenotypes, not just the aggregated phenotypes. We then average these spatial features across nodes within the dominant NC to obtain representative spatial characteristics.

Step 5: Linking Spatial Features to Survival We associate the averaged spatial features from dominant NCs with the survival probability at the latest time point in the Kaplan-Meier curve. Additionally, we compute the BR ratio (the proportion of BR images) for each patient in valid PCs.

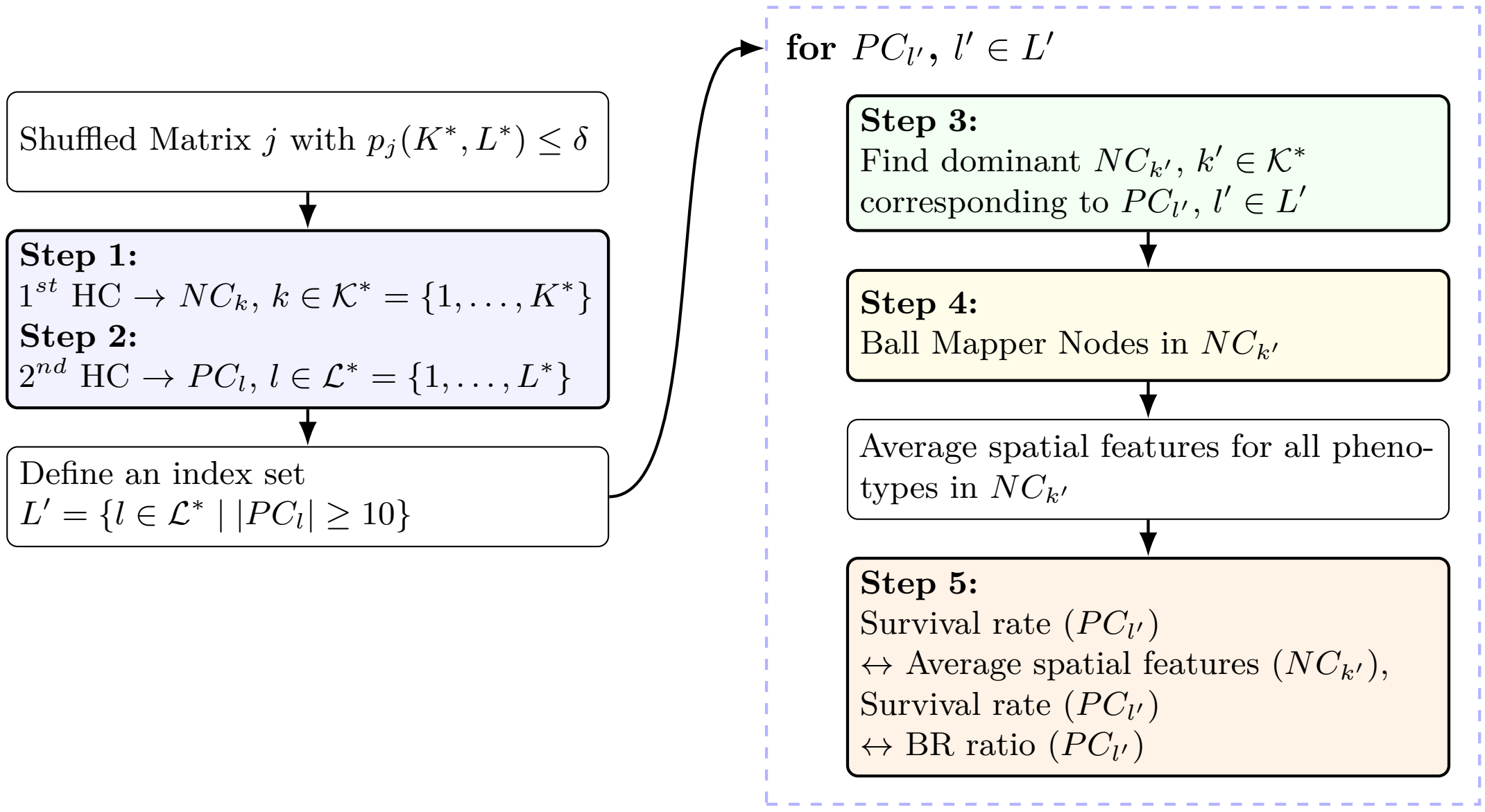

Fig. S4: Conceptual workflow for linking survival outcomes to spatial features. For shuffled matrix j with $p_j(K^*, L^*) \leq \delta$, clustering produces valid patient clusters $PC_{l'}$ (with $|PC_{l'}| \geq 10$). For each $PC_{l'}$, a dominant node cluster $NC_{k'}$ is identified (Step 3), Ball Mapper nodes are extracted (Step 4), and averaged spatial features are computed. These and BR ratio outcomes are then linked to survival rate (Step 5).

Step 6: Aggregation Across Multiple Shuffled Matrices We repeat the above procedure for all shuffled feature matrices satisfying $p_j(K^*, L^*) \leq \delta$; see the subsection *Spatial interaction patterns in $s\mathcal{DD}$ space associate with patient survival* in the main text. This generates multiple associations between spatial features (of all phenotypes) and survival probabilities (or BR ratios). We aggregate these results to examine relationships between averaged spatial feature values and survival outcomes.

Note that we use dominant NCs rather than averaging all features of images per patient because patient-level feature can be heterogeneous. Simple averaging across all features of a patient may be skewed by a few point clouds exhibiting spatial patterns different from the majority. By identifying the dominant NC for each patient group, we capture the predominant spatial patterns more robustly.

S6 Overview of the End-to-End Analytical Framework

This section provides a schematic overview of the end-to-end analytical framework proposed in this study (Fig. S5). For each point cloud, we compute distance functions from the boundaries of Topological Malignant Region (TopMR) and density functions from malignant cell distributions. These paired values at immune cells form the signed distance-density ($s\mathcal{DD}$) space, from which spatial features are extracted to build a comprehensive feature matrix. To explore diverse perspectives of the high-dimensional features, we repeatedly shuffle the matrix, selecting statistically significant shuffled matrices via optimal cluster identification. From graphs of Ball Mapper, we further analyze through a two-stage hierarchical clustering (HC), followed by Kaplan-Meier (KM) survival analysis on identified patient clusters. All valid patient clusters are collected by corresponding survival probabilities and spatial features from selected matrices. Survival probabilities are divided into two survival groups, with spatial features averaged within each group to identify associations between spatial interaction patterns and clinical outcomes.

S7 Clinical Characteristics of the Study Cohort

The study cohort consists of 78 diffuse large B-cell lymphoma (DLBCL) patients. Clinical and demographic characteristics including age, gender, treatment assignment, WHO performance status, MSKCC prognostic score, and number of lesions are presented in Table S2.

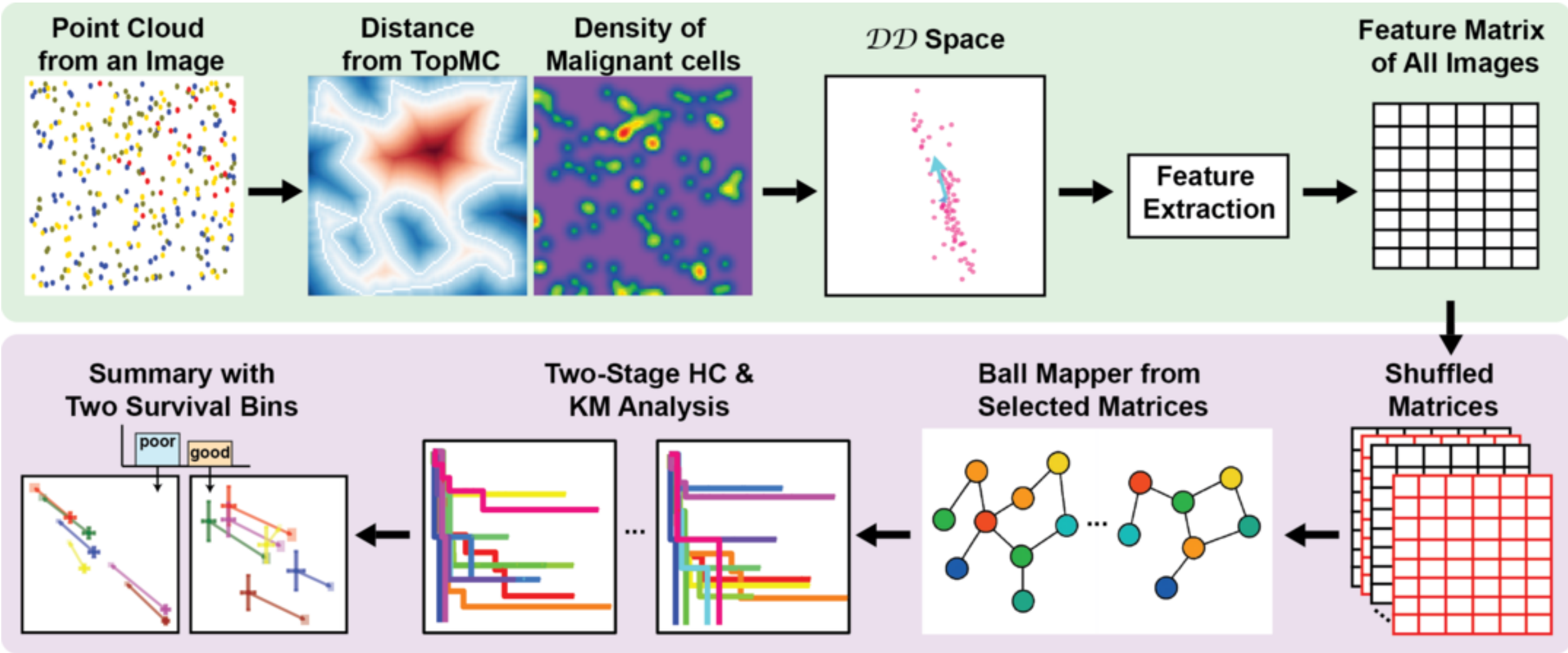

Fig. S5: Schematic overview of the proposed analytical framework.

Table S2: Cohort overview

Demographics		Treatment & Status		Prognosis	
# samples	78	Treatment arm		MSKCC score	
Age	59.9 (26–70)	MBVP	43 (55%)	1	8 (10%)
Gender		R-MBVP	35 (45%)	2	45 (58%)
Male	44 (56%)	WHO status		3	25 (32%)
Female	34 (44%)	WHO-0	13 (17%)	# lesions	2.47 (1–9)
		WHO-1	38 (49%)		
		WHO-2	11 (14%)		
		WHO-3	16 (21%)		

S8 Sensitivity Analysis of Ball Mapper Parameter ϵ

Stability of the Ball Mapper graph under different ϵ values has been explored through visual comparison; see Figures 7–8 in [Ref. S1]. Here, we complement this with a quantitative analysis. In the main text, we fix $\epsilon_0 = 4.5 \times 10^{-2}$ so that a large connected component may dominate the graph structure of the combined TR+BR feature matrix. To verify that this choice yields a stable graph and to characterize how the graph changes across a range of ϵ values, we perform a sensitivity analysis using 21 values, uniformly spaced over $[0.5\epsilon_0,\ 1.5\epsilon_0]$. We apply this analysis to three feature matrices: TR+BR (972 images $\times$ 18 features), TR-only (687 images), and BR-only (285 images). In the following, each row of a feature matrix (i.e., each image) is referred to as a *sample*.

We evaluate the sensitivity of the Ball Mapper graph at three levels: graph structure, component dominance, and image-level membership stability.

Graph Structure: For each ϵ, we count the number of nodes and edges to check that the Ball Mapper algorithm behaves consistently with its construction on our data. As shown in the top row of Fig. S6, the number of nodes decreases smoothly and monotonically across the tested range in all three feature matrices. This reflects that increasing ϵ produces larger balls that cover more samples, requiring fewer landmarks. The number of edges generally decreases with increasing ϵ but shows a slight increase near ϵ_0 in the TR{+}BR and TR matrices. This coincides with the formation of a dominant connected component (see below): as ϵ increases through this range, balls from previously separate components begin to overlap, connecting them and creating additional edges. The BR-only matrix exhibits more variability in edge count, which is expected given its smaller sample size.

Dominant Component Formation: To assess whether a connected component (i.e., a maximal set of nodes connected by paths of edges) contains the majority of samples, we compute the *largest component fraction* (LCF).

Let $\mathcal{C}_1, \ldots, \mathcal{C}_m$ denote the connected components of the Ball Mapper graph at a given ϵ. Each component

$\mathcal{C}_l$ is a set of nodes. For each node v, let B_v denote the set of samples assigned to that node. Since Ball Mapper produces an overlapping cover where a single sample can belong to multiple nodes, we define the set of unique samples in component $\mathcal{C}_l$ as

$$U(\mathcal{C}_l) \;=\; \bigcup_{v \in \mathcal{C}_l} B_v, \tag{6}$$

where the union ensures that samples shared across multiple nodes within the same component are counted only once. The largest component fraction is then defined as

$$\mathrm{LCF}(\epsilon) \;=\; \frac{\max_l \left| U(\mathcal{C}_l) \right|}{n}, \tag{7}$$

where n is the total number of samples in the feature matrix.

In the middle row of Fig. S6, we present LCF graphs over ϵ for feature matrices of TR+BR, TR-only, and BR-only images. For the case of TR+BR, the LCF rises from approximately 0.68 at $\epsilon = 0.043$ to 0.94 at $\epsilon_0 = 0.045$, showing that ϵ_0 is the value at which a single component begins to dominate the graph, as mentioned in the main text. This transition is consistent with the edge count peak observed in the top row: as separate components merge into one, new edges form between their nodes. Once the dominant component is established ($\epsilon \geq \epsilon_0$), the LCF remains above 0.90 in the feature matrix of TR+BR images, indicating that the dominant component persists when $\epsilon \geq \epsilon_0$.

Since ϵ_0 is determined from the feature matrix of TR+BR images, we also examine how the same value behaves on the TR-only and BR-only matrices separately. The TR-only matrix shows a similar transition, reaching LCF ≈ 0.85 at ϵ_0. The BR-only matrix remains at LCF ≈ 0.38 throughout the tested range. Because this value does not significantly change with the range of ϵ, the absence of a dominant component in BR is a property of the data rather than a consequence of the ϵ choice.

Co-Membership Stability: The Ball Mapper output depends on the parameter ϵ: as ϵ varies, nodes split, merge, and reorganize, and both the node set and the sample-to-node assignment change accordingly. To claim that the output is not an artifact of a particular choice of ϵ, one must specify which aspect of the output is to be declared stable. The grouping of samples, which samples are placed together, is also an intrinsic property of the graph and we therefore assess stability at this level.

Setting $\epsilon^{(k)} = \epsilon_0 + k\Delta\epsilon$ with $\Delta\epsilon = \epsilon_0/20$ and $k = -10, -9, \ldots, 10$, at each level k, let $V^{(k)}$ denote the node set produced by Ball Mapper and $B_v^{(k)}$ the samples assigned to node $v \in V^{(k)}$ (the same B_v as in Eq. (6), now indexed by k). A direct comparison of $V^{(k)}$ and $V^{(k+1)}$ is ill-posed. Node indices carry no intrinsic meaning, so any stability measure should be invariant under relabeling; and because nodes split and merge with ϵ, the cardinalities $|V^{(k)}|$ and $|V^{(k+1)}|$ generally differ, so no bijection between the two node sets exists. Any node-to-node correspondence must therefore be imposed by an external matching rule that is not determined by the data, and the resulting stability measure would reflect the arbitrariness of this rule as much as the Ball Mapper output itself.

The binary relation “samples i and j share at least one node” avoids this problem. At each ϵ, the set of such pairs is determined directly from the cover $\{B_v^{(k)}\}$ without invoking any correspondence between $V^{(k)}$ and $V^{(k+1)}$. It is invariant under node relabeling, since permuting node indices leaves the collection of pairs (i, j) co-assigned to some node unchanged. Furthermore, when ϵ varies and a set of samples migrates together as a subset of some node at both levels, all pairwise co-memberships within that set are preserved by construction; only those splits and merges that separate previously co-assigned samples alter the co-membership set. This relation therefore captures the grouping information at a level that is insensitive to node relabeling and to reorganizations that preserve which samples are grouped together, and we adopt it as the basis for stability assessment. Formally, the co-membership set at level k is

$$S^{(k)} = \big\{(i,\, j) \;\big|\; i < j,\; \{i, j\} \subseteq B_v^{(k)} \text{ for some } v \in V^{(k)}\big\}, \tag{8}$$

and the grouping distance between two levels is quantified by the Jaccard similarity

$$J\left(S^{(k)}, S^{(l)}\right) = \frac{\left|S^{(k)} \cap S^{(l)}\right|}{\left|S^{(k)} \cup S^{(l)}\right|}, \tag{9}$$

where $J = 1$ indicates identical grouping structure and $J = 0$ completely disjoint co-member pairs.

We compute J for adjacent ϵ values rather than for distant pairs such as $J(S^{(-10)}, S^{(10)})$. A single distant comparison reports only the total change accumulated across the range of ϵ and therefore cannot

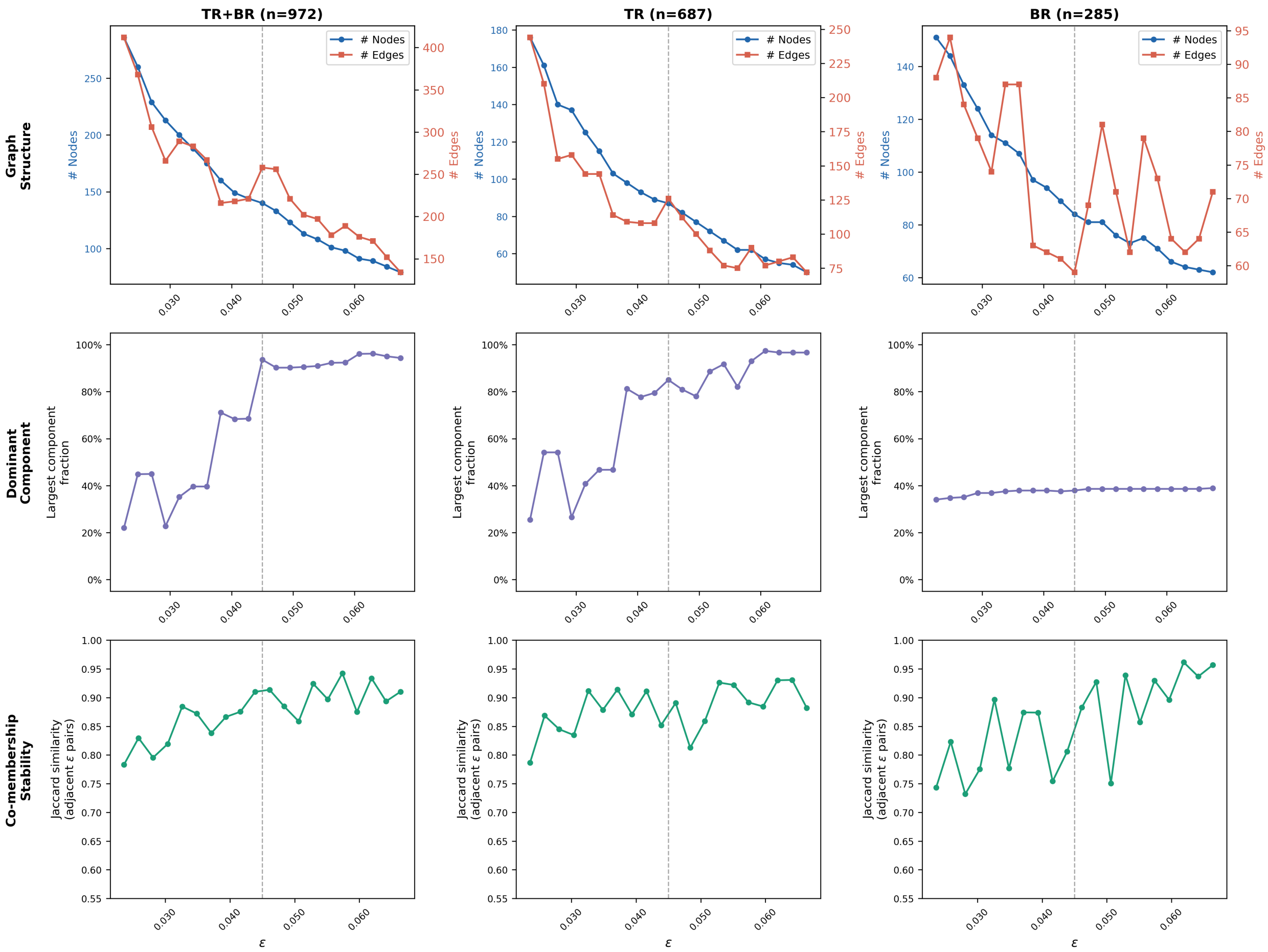

Fig. S6: Sensitivity analysis of Ball Mapper parameter ϵ. Each column corresponds to one of three feature matrices: TR+BR ($n = 972$), TR only ($n = 687$), and BR only ($n = 285$). The dashed vertical line marks $\epsilon_0 = 4.5 \times 10^{-2}$. **Top row**: Number of nodes (left axis, blue) and number of edges (right axis, red) as a function of ϵ. **Middle row**: Largest component fraction (LCF, Eq. (7)), i.e., the proportion of samples in the largest connected component with respect to total number of samples. **Bottom row**: Co-membership Jaccard similarity (Eq. (9)) between adjacent ϵ values.

distinguish between two scenarios: (i) a smooth gradual drift of grouping across the range, and (ii) a sharp reorganization at one critical ϵ surrounded by otherwise stable regions. The adjacent-pair profile $J(S^{(k)}, S^{(k+1)})$, $k = -10, \ldots, 9$, separates these scenarios and allows any local transition in ϵ to be localized.

The bottom row of Fig. S6 reports $J(S^{(k)}, S^{(k+1)})$ across the three feature matrices. The similarity remains consistently high: 0.78–0.94 for TR+BR (mean 0.88), 0.79–0.93 for TR (mean 0.88), and 0.73–0.96 for BR (mean 0.86). Each increment of $\Delta\epsilon$ thus preserves 78–94% of the pairwise grouping structure. No adjacent pair shows a sharp drop, and in particular no value in the neighborhood of the operating point ϵ_0 deviates from this plateau. The sample grouping produced by Ball Mapper at ϵ_0 therefore lies within a locally stable regime of the parameter.

References

[Ref. S1] Paweł Dłotko, Davide Gurnari, , and Radmila Sazdanovic. Mapper–type algorithms for complex data and relations. JCGS, pages 1–14, 2024.
